# Epidemiology, control, and prevention of Newcastle disease in endemic regions: Latin America

**DOI:** 10.1007/s11250-019-01843-z

**Published:** 2019-03-15

**Authors:** A. E. Absalón, Diana V. Cortés-Espinosa, E. Lucio, P. J. Miller, C. L. Afonso

**Affiliations:** 1 Vaxbiotek, S.C. San Lorenzo 122-7, 72700 Cuautlancingo, Puebla Mexico; 2 Instituto Politécnico Nacional, CIBA-Tlaxcala, Carr. Est. Santa Ines Tecuexcomac-Tepetitla Km. 1.5, 90700 Tepetitla, Tlaxcala Mexico; 3 Boehringer Ingelheim Animal Health, PO Drawer 2497, Gainesville, GA 30503-2497 USA; 4 Department of Population Health, College of Veterinary Medicine, The University of Georgia, 953 College Station Road, Athens, GA 30602 USA; 5 Exotic and Emerging Avian Viral Disease Research Unit, Southeast Poultry Research Laboratory, United States National Poultry Research Center, USDA/ARS, Athens, GA 30605 USA

**Keywords:** Newcastle disease, Epidemiology, Endemic, Vaccines, Evolution, Disease control

## Abstract

Newcastle disease (ND) infects wild birds and poultry species worldwide, severely impacting the economics of the poultry industry. ND is especially problematic in Latin America (Mexico, Colombia, Venezuela, and Peru) where it is either endemic or re-emerging. The disease is caused by infections with one of the different strains of virulent avian Newcastle disease virus (NDV), recently renamed *Avian avulavirus 1*. Here, we describe the molecular epidemiology of Latin American NDVs, current control and prevention methods, including vaccines and vaccination protocols, as well as future strategies for control of ND. Because the productive, cultural, economic, social, and ecological conditions that facilitate poultry endemicity in South America are similar to those in the developing world, most of the problems and control strategies described here are applicable to other continents.

**Electronic supplementary material:**

The online version of this article (10.1007/s11250-019-01843-z) contains supplementary material, which is available to authorized users.

## General introduction: Newcastle disease (ND)

Poultry farming is one of the most important livestock producing activities in the world because it supplies low-cost animal protein. Nevertheless, since its beginning, the main threat to the industry has been the occurrence of diseases that decrease production. One of the most common and detrimental avian viral diseases affecting poultry production is Newcastle disease (ND), caused by infections with virulent viruses from the genus Avulavirus and species *avian avulavirus 1,* commonly known as Newcastle disease virus (NDV) and abbreviated as avian paramyxovirus 1 (APMV 1) (Mayo 2002; Afonso et al. 2016; Amarasinghe et al. 2017, 2018). The disease is highly contagious, and without an adequate control strategy, causes high morbidity and mortality rates in naïve or poorly vaccinated chickens, as well as drops in egg production in well-vaccinated layers (Alexander et al. 2004; Perozo et al. 2008; Miller et al. 2010). The virus is capable of infecting at least 236 bird species, including the majority of wild and domestic bird species (Kaleta and Baldauf 1988), and infections of birds from at least 20 of the 26 Orders in the Clements classification system for modern birds have been reported (Miller and Koch 2013).

## Molecular epidemiology of virulent Newcastle disease virus

Based on genetic characteristics, NDV has been classified into class I and class II viruses. Class I NDV have been isolated predominantly from wild birds, are mostly of low virulence, and their presence is only rarely reported in poultry species. Classification of class II NDV strains has been thoroughly reviewed elsewhere (Dimitrov et al. 2016c). Only a handful of low virulent isolates of viruses of class I genotype I have been reported in Colombia (KJ865697.1, KJ865703.1, KJ865704.1, KJ865705.1, KJ865711.1) and Mexico (KC808493.1, KC808494.1) during 2009 to 2011. All NDV strains of low virulence are negative when tested with real-time PCR fusion assays designed to detect virulent NDV (Wise et al. 2004), may not be tested any further, and thus, be underreported. Furthermore, because most chickens are vaccinated with vaccines formulated with NDV of low virulence, samples that are matrix positive (confirming NDV) and fusion negative (confirming low virulence) may be assumed to be vaccine strains rather than wild-type field strains of low virulence (Wise et al. 2004).

As the molecular epidemiology of NDV strains of low virulence is poorly understood, and the presence those viruses in poultry is not always being reported, here, we will focus on the epidemiology of NDV strains that are capable of causing disease. Despite the limitation that poultry infections with virulent NDV in Latin America not always being reported by farmers, progress has been made in recent years to identify the diversity of NDV strains circulating across the continents. Furthermore, a comparison of these strains with viruses circulating worldwide has resulted in a better understanding of the negative impact on production incurred upon the introduction of novel virulent NDV strains (Dimitrov et al. 2016c).

Because of their worldwide distribution and the high mobility of their avian hosts, virulent NDV strains from any continent have the potential to be introduced into Latin America. In the past, evidence of introductions from different continents, followed by further evolution of the viruses at local sites, has been documented across Latin America (Diel et al. 2012c; Perozo et al. 2012). There is also evidence that the different virulent genotypes do evolve independently (Miller et al. 2009b) in different geographic locations with clearly distinct nucleotide and amino acids differences (Dimitrov et al. 2016b). Furthermore, new viral genotypes can emerge over time in isolated locations and have a negative impact on poultry farming (Courtney et al. 2013; Snoeck et al. 2013). Virulent forms of class II NDV are frequently reported in chickens and pet species; however, spillovers into wild birds do occur (Cardenas Garcia et al. 2013; Ayala et al. 2016). In addition, specific genotypes of virulent NDV are also maintained in wild birds, such as pigeons and cormorants (genotype VI) (Diel et al. 2012b; Sabra et al. 2017; He et al. 2018).

**Fig. 1:**
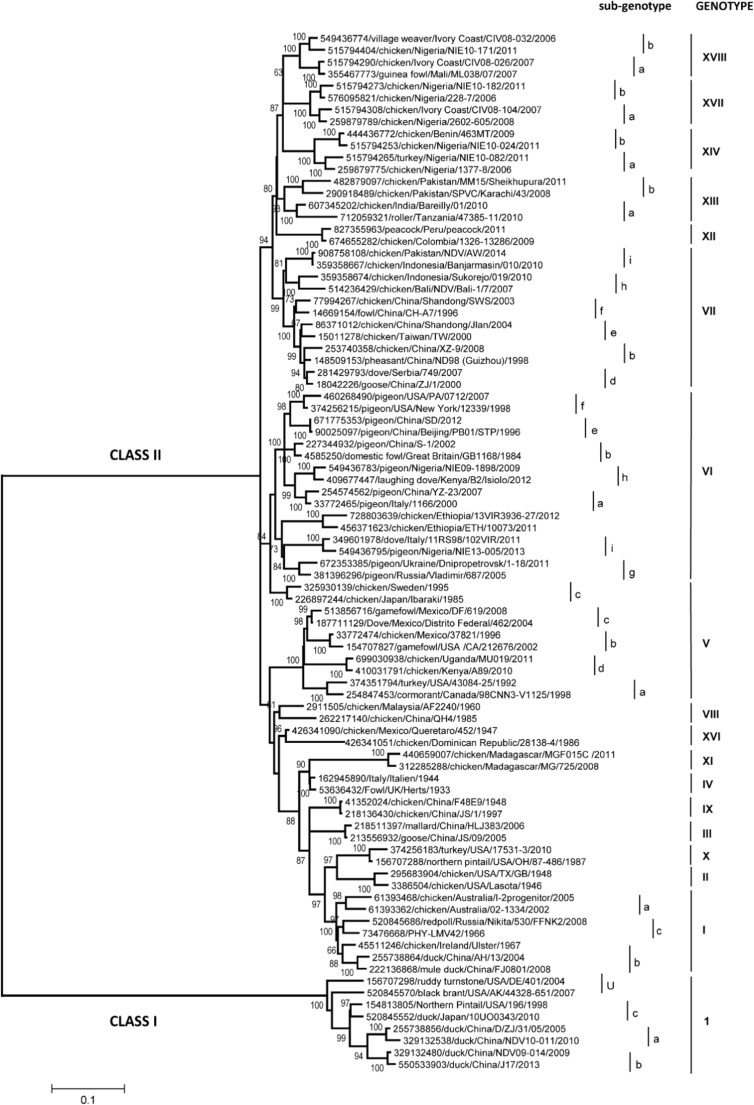
Phylogenetic tree constructed using the complete nucleotide sequences of the fusion gene of representative isolates of avian Avulavirus class I and class II showing the evolutionary relation of 18 current genotypes of Newcastle disease virus, including the subgenotypes of genotype V (Dimitrov et al. 2016c)

The genetic diversity of class II NDV probably originates in the intrinsic errors of the viral polymerase during genome replication. These alterations are believed to create a large number of genetic variants known as quasispecies, on which natural forces act to select determined characteristics of the NDV genome. With the exception of a few sites mapped on selected isolates (Dortmans et al. 2011), the roles of the innumerable mutations existing on circulating viruses on the pathogenesis and host range are still unknown. Overall, there is a greater genetic variability within class II viruses than within class I; currently with 18 class II genotypes (Fig. 1, Dimitrov et al. 2016c) that are classified based on the variability of protein F or the whole genome (Diel et al. 2012a; Courtney et al. 2013; Snoeck et al. 2013). A genome of a genotype V NDV has been identified that contains evidence of viral genetic recombination (Miller et al. 2009b); however, there has been no evidence that the progeny from this strain has persisted or facilitated the development of a new genotype. While genetic recombination between viruses has also been suggested for some other strains (Han et al. 2008; Zhang et al. 2010), the phenomenon has been questioned (Afonso 2008; Song et al. 2011), and its role as an evolutionary mechanism of NDV is yet to be confirmed. Recent studies have demonstrated that the proposed recombinant viruses were instead mixed infections (Song et al. 2011).

### A. Virulent NDV in the Caribbean, Central, and North America

Since the 1970s, viruses of genotype V have been frequently reported in Central America including the countries of Honduras (2000 and 2007), Nicaragua (2001), and Belize (2008) suggesting that this genotype may have become established in some unknown reservoir (Susta et al. 2014; Brown et al. 2018). In North America, the frequent outbreaks in Mexico resulted in the isolation of virulent NDV strains from genotype V in 1988, 1996, 2000, 2001, 2005, and 2008 to 2011 (Absalón et al. 2014). On both Central and North American continents, genotype V strains are the predominant NDV found, with reports of isolations in both wild birds and poultry (Cardenas Garcia et al. 2013; Susta et al. 2014). Regular isolations of virulent NDV from genotypes V and VI have been reported in the USA from cormorants and pigeons, respectively (Diel et al. 2012b; He et al. 2018).

The genetic variation of genotype V viruses is quite significant, making it possible to identify three subgenotypes: Va, Vb, and Vc (Fig. 1) (Absalón et al. 2014; Susta et al. 2014; Dimitrov et al. 2016c). The analyses of nucleotide sequence of the fusion protein (F) genes provide evidence of genetic differences between the three subgenotypes. In subgenotypes Vb and Vc isolated from commercial birds, differences between 4 and 7% were found; however, the differences between subgenotypes of commercial birds (Vb and Vc) compared with the subgenotype of wild birds (Va) are between 7 and 9%. Viruses of genotypes Vb and Vc are velogenic viscerotropic, causing gross lesions in the visceral organs, while viruses of genotype Va are either velogenic neurotropic or mesogenic, causing minimal gross lesions. In Central America (Belize), viruses within subgenotype Vb have evolved separately, forming their own clade that is distinct from the Vb strains isolated in Mexico (Susta et al. 2014). The other Vb clade corresponds to strains related to the La Laguna outbreak in Mexico in 2000 (Merino et al. 2009) and the 2002 outbreak in California, USA (Pedersen et al. 2004); meanwhile, subgenotype Vc contains strains of the outbreak in 2004–2005 (Absalón et al. 2012a). As recently as May 2018, the re-emergence of virulent viruses of genotype Vb has been observed in backyard poultry in the USA www.aphis.usda.gov/animalhealth/vnd. Finally, subgenotype Va strains group with the strains isolated from wild birds in the USA (Diel et al. 2012b). This confirms that genotype V continues to circulate and evolve rapidly despite the ongoing vaccination campaigns in Mexico, Central, and South America. It is possible that, as in the case of the avian influenza virus (Lee et al. 2004), vaccination provides antigenic pressure for the evolution of field viruses in broiler chickens; however, there are no laboratory studies to date that demonstrate this phenomenon (Perozo et al. 2008).

In the Dominican Republic, an outbreak was reported in 2008 (Courtney et al. 2013). The virus NDV-DR499-31/08 was isolated from specimens collected in 2008 during routine surveillance of an apparently healthy flock of chickens after an avian influenza virus was detected the previous year. A second isolate was collected as a result of the existence of clinical signs in commercial hens from a flock of 80,000 that was experiencing an increase in the mortality rate (∼3%) (Courtney et al. 2013). After virus isolation, the complete genome and the full fusion genes of these isolates were sequenced and found to be related to older virus circulating in the country (chicken/Dominican Republic/28138-4/1986 and chicken/Mexico/Queretaro/452/1947) (Courtney et al. 2013). Genetically, the new Dominican isolates and their ancestors were clearly distinct from all other currently known isolates of NDV and from any other available sequence existing in GenBank; therefore, they were classified as members of a new genotype (genotype XVI). The lack of any reported isolation of NDV related to this group in the Dominican Republic between 1986 and 2004 suggests that virulent NDV strains of this genotype may have evolved unnoticed for 22 years in the Caribbean (Courtney et al. 2013). The NDV-DR499-31/08 strain had an intracerebral pathogenicity index (ICPI) value of 1.88, and through the sequencing of the fusion cleavage site, multiple basic amino acids and a phenylalanine at position 117 were identified, indicating this isolate to be highly virulent (Courtney et al. 2013), which makes the unnoticed existence of virulent viruses in the country more puzzling. However, the existence of a strong phylogenetic relationship between the 2008, 1986, and 1947 viruses clearly links them and suggests that virulent NDV strains may have evolved in unknown reservoirs in the Caribbean.

### B. Virulent NDV in South America

Most reported isolations corresponded to virulent viruses, with the earliest viruses being reported in Argentina in 1970 (AY734534) and Brazil in 1953 (Santos et al. 1954). In Brazil. the subgenotype Vb was detected in 1975 (KJ123642) (Fernandes et al. 2014), and it continues to circulate in the 90s (AY 175695, AY 175648, AY 175649) (Aldous et al. 2003). The last detection in backyard poultry was reported in 2006 in three distinct Brazilian states (RS, AM, and MT) (OIE 2006). In 2014, a genotype VI h (KX0907024) closely related to VI h in Argentina in the 1990s had been found causing disease in feral pigeons in Brazil (Souza et al. 2018). In Peru, the NDV strain poultry/Peru/1918-03/2008 was isolated from swab samples during an outbreak of ND affecting domestic poultry flocks in the region of Pachacutec, Arequipa, Peru, in 2008 (Diel et al. 2012c). Clinical and pathological characterization and ICPI studies conducted on chickens revealed that these viruses presented typical characteristics of virulent velogenic viscerotropic NDV strains. Vaccination studies revealed that the inactivated formulation of the classical vaccine strain LaSota was sufficient to prevent clinical disease upon challenge; however, it did not prevent infection with or virus secretion of the virulent challenge strains (Diel et al. 2012c Miller et al. 2013). Although the live LaSota vaccine was not tested, there is no indication that this vaccine would have failed to protect the original isolates. The complete genome sequence characterization revealed the existence of large genomic and amino differences between the Peru isolate and known sequences that clearly distinguish this isolate from previous viruses of typical NDV genotypes. Alignment of the complete genome sequences revealed that the Peru isolates have low nucleotide identity with the vaccine strains Ulster/67 (84.8%; genotype I) and LaSota/46 (82.5%; genotype II) (Diel et al. 2012c). During 2010 and 2011, five other NDV isolates were obtained from geese in the live-bird markets in China (Diel et al. 2012a). The amino acid sequence identities of the (F) and hemagglutinin-neuraminidase (HN) proteins among these five isolates ranged from 99.1 to 100% and 99.1 to 99.6%, respectively, and their intracerebral pathogenicity index (ICPI) values ranged from 1.74 to 1.93 in 1-day-old chickens (Diel et al. 2012a). Phylogenetic analysis based on the complete nucleotide sequences of the F and HN genes classified these isolates and the Peru strain (poultry/Peru/1918–03/2008) into a new genotype designated genotype XII (Diel et al. 2012a). Additionally, complete genome analysis of strains, goose/GD1003/2010 and goose/GD450/2011, showed that the highest genetic identity among existing GenBank sequences corresponded to the poultry/Peru/1918–03/2008 strain (GenBank accession number JN800306) (Diel et al. 2012a). Viruses of this genotype continued to be isolated in Peru until 2017.

Outbreaks of Newcastle disease have been detected in Colombia and Venezuela over the last 10 years. The NDV strain chicken/Venezuela/611/2008 (VEN-611) was obtained in May of 2008 from a commercial pullet flock presenting high mortality rates and clinical signs of ND (Perozo et al. 2012). Biological pathotyping showed a mean embryo death time of 50 h and an ICPI of 1.86 (Perozo et al. 2012). Sequence-based phylogenetic analysis demonstrated that this NDV belonged to class II subgenotype VIId, most often found in Asia and Africa (Dimitrov et al. 2016c), representing the first report of the presence of this genotype in the continent of South America (Perozo et al. 2012). Subgenotype VIId NDV isolates have also been reported in Europe (Dimitrov et al. 2016a). In 2009, Newcastle disease viruses from genotype XII were isolated from outbreaks in Colombia, suggesting that the viruses from Peru have moved into the northern part of the South American continent (Berhane et al. 2017). Furthermore, the significant genetic differences between NDV of all genotypes from Peru, Colombia, and Venezuela from those isolated from Mexico and Central America suggest that independent introductions have occurred (Dimitrov et al. 2016c).

## Control strategies

### A. Limitations of current control strategies

As there is no treatment for ND, the culling of infected birds combined with strict biosecurity and aggressive vaccination protocols are the most suitable measures to control outbreaks. However, in most Latin American countries, there are no strong government-sponsored programs to support eradication through culling, and the most cost common alternative is the use of aggressive vaccination programs. Outbreaks worldwide have been attributed to a multitude of causes including a lack of biosecurity, deficient vaccines and vaccination programs, antigenic variation, inhibition of live vaccines by maternal antibodies, short duration of the immune response, and immune suppression (Chumbe et al. 2017; Dimitrov et al., 2017a, b). However, the underlying problem across Latin America appears to be the existence of reservoirs of viruses ready to cause disease in the event of any deficiency in any of the control strategies. Unfortunately, biosecurity and vaccinations alone have not been sufficient to eliminate the circulation of virulent NDV strains, which remain endemic and infect poorly vaccinated flocks often resulting in high levels of mortality (Miller et al. 2009a; Dimitrov et al., 2017a, b).

The NDV strains of low virulence (LaSota, Hitchner B1, Ulster, VGGA, among others) employed most commonly as seed strains for live, and inactivated vaccines are applied worldwide (Dimitrov et al., 2017a, b). These vaccines were originally isolated between 30 to 60 years ago and are classified within class II as genotypes II or I (Diel et al. 2012a). Vaccine producers justify the continued use of these vaccines, despite the diversity of virulent genotypes reported, because all NDV strains are grouped into one serotype. This means that under laboratory conditions, a vaccine made from any strain or genotype is capable of inducing humoral immunity to prevent clinical signs and mortality against a highly virulent challenge (Liu et al. 2003; Miller et al. 2007, 2009a; Dimitrov et al., 2017a, b). However, there are additional components involved to consider, such as cellular immunity, which is not defined by serotype.

While NDV strains used in commercial vaccines are capable of preventing disease and death when they are properly administered to healthy specific pathogen free (SPF) chickens that have no maternal antibodies to NDV under laboratory conditions, they do not prevent replication and shedding of virulent challenge virus into the environment and into eggs (Miller et al. 2007, 2009a; Sa e Sá et al. 2016). Furthermore, laboratory experience does not translate well into field outcomes, and field flocks with well-vaccinated birds often present with more moderate clinical disease, showing considerable reductions in weight per bird while shedding into the environment large amounts of virulent NDV in their feces and oral secretions (Miller et al. 2007; Rue et al. 2011; Absalón et al. 2012a). In addition, we have recently demonstrated that in well-vaccinated animals repeated challenges do not affect the protection inferred by the LaSota NDV vaccine (Taylor et al. 2017). Thus, virulent NDV strains continue to re-infect populations of broiler chickens and laying hens despite intense vaccination programs (Perozo et al. 2008).

The persistence of virulent NDV in the flocks and in the environment is likely one of the causes of slow weight gains in broilers and decreased quality in egg layers. Decreased egg quality is seen as amorphous (oddly shaped), smaller eggs with rough, thin, and whiter “bleached” shells and poor-quality contents (whites and yolks) (McFerran and McCracken 1988; Rao et al. 2002; Bwala et al. 2011; Bertran et al. 2017). Reduced egg laying and gross lesions including marked atresia, resorption, hemorrhage of ovarian follicles, and rupture of the yolks that infiltrated into the abdominal cavities are observed in laying hens infected with virulent NDV strains (Rao et al. 2002; Bwala et al. 2011; Igwe et al. 2018). It is also suspected that the vaccination of laying hens with live NDV vaccines may negatively affect egg laying by resulting in small decreases in productivity; however, this observation has not been proven. The greater insults to egg production, decreased egg quantity and quality, are known sequelae of infections with virulent NDV, even in well-vaccinated birds (Rao et al. 2002; Mumma et al. 2006; Bwala et al. 2011; Absalón et al. 2012a).

**Fig. 2:**
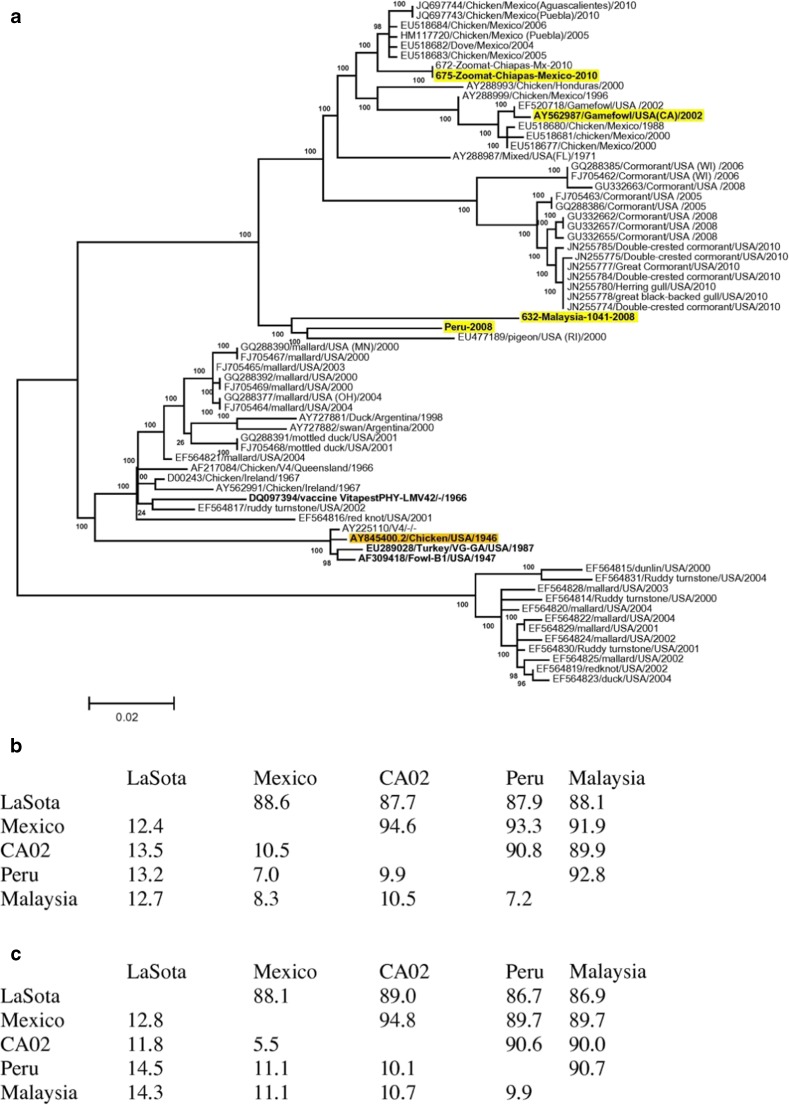
**a** Molecular phylogenetic analysis of Newcastle disease isolates by maximum likelihood method. Analysis of the full fusion protein was performed as described in the section 2. The tree is drawn to scale, with branch lengths measured in the number of substitutions per site. The analysis involved 68 amino acid sequences. All positions containing gaps and missing data were eliminated. There was a total of 550 positions in the final dataset. Evolutionary analyses were conducted in MEGA5. Virulent viruses utilized in vaccination experiment are highlighted in yellow (virulent viruses) and vaccine viruses are highlighted in orange. **b** Fusion protein amino acid differences between the LaSota and B1 vaccine viruses compared to selected vaccine and virulent challenge viruses (Miller et al. 2013b)

Part of the difficulties of vaccines and vaccination programs in preventing viral replication may be attributed/accredited to antigenic differences. The effect of antigenic differences is likely to increase the circulation of viruses in farms and the surroundings, thus leaving poorly vaccinated or immune suppressed animals susceptible to disease. As antigenic differences are not easily determined, the analysis of evolutionary relatedness between vaccine viruses and circulating strains has relied on comparisons of the predicted amino acid sequence of the genes that encodes the fusion protein and the HN (Hu et al. 2009; Miller et al. 2010; Absalón et al. 2012a). There is noticeable divergence (Fig. 2) in the sequence of the predicted protein of the two NDV surface antigens, HN and F, among genotype II vaccine strains and the field viruses of genotypes V, VI, VII, and XIII circulating in poultry farms (Hu et al. 2009; Absalón et al. 2012a; Diel et al. 2012a; Miller et al. 2013; Susta et al. 2014). It is worth emphasizing that the most frequent genotypes affecting poultry farms in Latin America correspond to genotypes V, VI, VII, XII, and XVI (Fig. 1) (Absalón et al. 2012a, 2014; Susta et al. 2014; Dimitrov et al. 2016c).

Over the last 25 years, multiple studies (Erdei et al. 1987; Aldous and Alexander 2001) have presented evidence of the existence of antigenic differences between strains of NDV using monoclonal antibodies. These antigenic differences enabled differential diagnosis between birds vaccinated with the LaSota vaccine strain and virulent field viruses causing outbreaks in poultry farms. Indeed, Erdei and collaborators (1987) identified monoclonal antibodies induced by the LaSota strain that do not recognize other viruses from among more than 300 lentogenic, mesogenic, and velogenic strains tested belonging to other genotypes (Erdei et al. 1987). Thus, it is possible that some of the epitopes of the antigenic proteins of the vaccine strain may induce antibodies that efficiently recognize all strains and also strain-specific antibodies (Erdei et al. 1987). Similarly, it has long been known that different hemagglutination inhibition titers are obtained depending on the NDV strain used as the antigen in the assay (Table 3) (Miller et al. 2013; Cardenas-Garcia et al. 2015).

Despite these differences, commercially available NDV vaccines are capable of efficiently preventing mortality and the severe signs of the disease in the face of a virulent NDV challenge in SPF birds in the absence of immune suppression, stressors, or management factors that in the field may affect the efficacy of vaccines (Nayak et al. 2012). Since small differences in amino acid sequences are known to affect the antigenic properties of proteins, there is room to increase the efficacy of vaccines by eliminating antigenic differences. The amino acid sequence of proteins F and HN from the LaSota strain diverge between 6 and 14% when compared with the sequences of proteins F and HN from strains representative of each of the 18 NDV genotypes (Fig. 2) (Miller et al. 2013; Dimitrov et al. 2016c). In order to improve protection, there is also a need to understand if these differences have an effect on the neutralizing antibody-antigen binding sites. That is, the strength of the bond (avidity) of the vaccine-induced antibodies to the antigenic proteins of the virulent challenge virus capacity to carry out the neutralization necessary to prevent the birds from being infected (Iorio and Bratt 1984; Erdei et al. 1987; Liu et al. 2003; Miller et al. 2007).

### B. Vaccines used in Latin America

Preventive programs are currently used in all Latin American countries using live and inactivated vaccines (Supplementary Table 1). It is of the highest importance to use not only the proper vaccines but also to adequately design the vaccination program that will give the best protective result in terms of clinical protection and in reducing the shedding of the virus in vaccinated flocks. Since Mexico is one of the Latin American countries with the longest presence of virulent NDV (Susta et al. 2014) and since ND vaccination programs are similar across Latin American countries with the presence of virulent NDV (all countries except Chile, Argentina, Brazil, Uruguay, and Costa Rica), we will describe here, in detail, the current vaccination practices used in Mexico.

Most broiler programs recommend at least three doses of vaccine; however, depending on the incidence of the virus, up to 4 to 5 vaccinations may be used (Supplementary Table 1). For broiler chickens, current practice includes vaccination as early as possible, which could include in ovo administration of vectored vaccines, such as recombinant HVT-NDV, or after hatching (at 1 day old) using live attenuated strain, such as B1. A booster vaccine is given 8–12 days later using either a live vaccine and/or inactivated vaccine. The inactivated vaccines are generally formulated using an oil adjuvant to induce a higher humoral response. Additional booster vaccines are also often applied at 4–5 weeks of age for longer-lived animals, like layers.

In Mexico, there is large diversity in the type of vaccines available for the prevention of ND, including live and inactivated oil emulsion formulations used alone or in combination with other viral, bacterial, or parasitic agents (Supplementary Table 1). Based on the information available by major trading houses with more than 1% of participation in the poultry market, there are at least 83 commercial vaccines (Supplementary Table 1). In total, 26 products contain the complete genome of NDV, of which 13 are live and 13 are inactivated oil emulsion vaccines.

The principal strain used in vaccine formulation is LaSota, but other strains of genotype II such as VG/GA, Clone 30, and B1 are also used. In addition, a genotype I (PHYML) vaccine and a recombinant genotype seed strain vaccine are also often used in Mexico. The rP05 vaccine contains a complete recombinant NDV of low virulence expressing the F and HN proteins of a genotype V NDV. Most of the oil emulsion type inactivated vaccines for the prevention of ND are mixed with two to four additional infectious antigens to decrease the number of times the birds have to be handled, resulting in less stress for the birds and decreased costs for vaccine administration. For example, in addition to the NDV genome, most vaccines also contain fowl adenovirus (usually serotype 4), infectious bronchitis virus, avian influenza virus (H5N2), and infectious bursal disease virus (Gumboro) (Table 1). Similar strategies are utilized for control of NDV across Latin America, and there are no significant differences in the approaches used in these countries. In Mexico and other countries, broilers have a vaccination calendar based on the number of live and inactivated vaccines they receive (Table 1). This extensive vaccination program is utilized with the principal goal of inducing humoral and cellular immune responses for the prevention of morbidity and mortality upon infection with a virulent NDV.

**Table 1:** Consensus vaccination calendar commonly used in broilers in Mexico

Age of birds	Kind of vaccine	Note
Week 1	Live	A live attenuated vaccine (as strain as B1) is administrated to chicks at day 1 of age by oral/eye drop. This vaccine depends on the presence of maternal antibodies. Usually all the chicks without maternal antibodies are vaccinated at day 1.
Week 2	Live /inactivated	Usually two vaccines, live and inactivated (in oil) are administrated at the same time (to avoid excessive management). This vaccination occurs between day 8 and 10.
Week 3	Live	At day 20–21 of age, a boost using a lentogenic strain follows. Indistinctly, VG/GA, LaSota, rP05 are used. This vaccine is administrated by way of drinking water.
Week 4	Inactivated	At day 28, a boost with an inactivated (in oil) vaccine is used.
Week 5	live	At day 35 of age, a boost with a live vaccine is administrated. Indistinctly, VG/GA, LaSota, rP05 are used. Particularly this boost is applied to birds that are kept in farms more than 48 days. This vaccine is administrated by way of drinking water*.

*Administration by aerosol (thick drop) is used occasionally in some chicken farms, when the birds in farms are healthy and the mortality by day is too low

Layers have extended vaccination programs to provide protection during their longer (compared with broilers) lifespan. For example, in Mexico during breeding (0 to 18 weeks of age), hens receive at least four vaccines against NDV (2 live/2 inactivated) (Table 2). When the hens are actively laying eggs after approximately 17 weeks, a schedule of vaccination using live vaccines and inactivated oil emulsions every 4 to 6 weeks is maintained (Table 2). In addition to vaccination with live NDV vaccines, live vectored vaccines formulated with *Meleagrid alphaherpesvirus 1*(also known as herpesvirus of turkey, HVT) expressing one of the genes encoding the antigenic proteins of NDV, mostly the fusion protein gene, are available and reviewed by Dimitrov et al. (2017a, b). While live NDV and live HVT-vectored vaccines stimulate the cellular and humoral immune responses, HVT-vectored vaccines also allow vaccinated birds to be differentiated from birds infected with field NDV strains (Sun et al. 2008). Furthermore, if a virus that infects the same host is used as vector, it can induce a response against the vector and against the heterologous antigenic protein, i.e., a bivalent vaccine (Bell 2001). There are numerous reports on vectors used for the expression of NDV antigenic proteins, among which are poxvirus and herpes virus.

**Table 2:** Consensus vaccination calendar commonly used during breeding in layers in Mexico

Age of birds	Kind of vaccine	Note
Week 1	Live vectored	Usually, chicks have a high level of maternal antibodies, and the vaccination is not necessary. However, vaccination with vectored vaccines based in HVT expressing the fusion protein of NDV is used.
Week 2	Live	At day 10–14 of age, a live vaccine using a lentogenic strain is administrated by drinking water. Indistinctly, VG/GA, LaSota, or P05 are used.
Week 3	No vaccine	Chicks are moved to breeding cages.
Week 4	Inactivated	At day 28, a boost with an inactivated (in oil) vaccine is administrated. Commonly, a unique vaccine containing multiple antigens is used. Almost never a monovalent NDV vaccine is used.
Week 5	No vaccine	
Week 6	No vaccine	
Week 8	No vaccine	
Week 8	Live	At day 55–60 of age, a live vaccine using a lentogenic strain is administrated by drinking water. Indistinctly, VG/GA, LaSota, or P05 are used.
Week 9	No vaccine	
Week 10	No vaccine	
Week 11	Live	Between weeks 11–12 of age, a live vaccine using a lentogenic strain is administrated to birds by drinking water. Indistinctly, VG/GA, LaSota, or P05 are used.
Week 12	No vaccine	
Week 13	No vaccine	
Week 14	No vaccine	
Week 15	Inactivated	During the 15th week of age, a boost with an inactivated (in oil) vaccine is administrated to birds. Usually, a unique vaccine containing multiple antigens is used.
Week 16	No vaccine	Hens are moved to production cages.
Week 17	No vaccine	Hens are moved to production cages.
Week 18	No vaccine	First week in production

**Table 3:** Pre- and post-challenge HI antibody titers (log_2_) to the homologous and heterologous antigens. Titers homologous between the vaccine and challenge virus are italicized. The post-challenge titers are in parenthesis to the right of the pre-challenge antibody titers (Miller et al. 2013b)

Serum vaccine groups	HI antigen						
	LaSota	Malaysia	Difference	Mexico	Difference	Peru	Difference
LaSota	*6.6*	5.5 (8.6)	3.06	5.7 (8.1)	2.37	6.5 (7.9)	1.48
Malaysia	6.0	*9.2 (10)*	*0.83*	6.4 (8)	1.56	6.8 (8.8)	2.00
Mexico	6.8	7.0 (10.3)	3.20	*8.6 (8.6)*	*0.00*	7.0 (7.9)	0.90
Peru	6.1	5.5 (7.4)	1.9	5.3 (7.3)	2.0	*6.2 (8.3)*	*2.06*

Usually, vaccines based on HVT as a vector expressing the NDV F protein (rHVT-NDV) are also included. HVT is a persistent apathogenic virus for chickens that induces long-term immunological protection, and is therefore, considered a good viral vector for use in poultry. In addition, HVT also induces protection against Marek’s disease virus, so these vaccines are capable of inducing protection against both viruses (Sondermeijer et al. 1993). The humoral response in the form of specific antibodies against NDV induced by live rHVT-NDV vaccine presents protective titers after vaccination when administered subcutaneously. This is one to 2 weeks later than the response induced by live and inactivated NDV vaccines, respectively. However, despite the delay in immunity, the benefit of the prolonged protection given by the rHVT-NDV vaccine is more important in longer-lived birds, such as laying hens and breeding hens (Rauw et al. 2010; Palya et al. 2012).

### C. Other preventive strategies

As discussed, the administration of NDV vaccines is the primary tool used to prevent clinical disease; however, the decrease of the amount of virulent NDV secreted into the environment is an additional benefit that is rarely considered in control strategies (Miller et al. 2013). NDV vaccines do not provide sterilizing immunity, and well-vaccinated birds can become infected without clinical signs (Miller et al. 2007, 2009a). However, levels of viral shedding can be reduced 100-fold with proper vaccination. Herd immunity exists when at least 85% of a flock has hemagglutination inhibition antibody titers equal to or greater than 8 to NDV (when using 8 hemagglutination (HA) units per 50 μl of antigen) and is essential for flock protection against ND (van Boven et al. 2008). The isolation of virulent NDV is significantly less likely to occur from flocks with herd immunity compared with flocks without herd immunity (Wiseman and Berman 2017), and herd immunity is important for preventing the spread of virulent NDV (Cornax et al. 2012). Unfortunately, it has also been demonstrated that in the field, herd immunity is not often achieved, and thus, it is important to carefully monitor flock immunity (Rehmani et al. 2015; Wajid et al. 2017).

As vaccinated flocks that have not yet reached appropriated level of protective immune responses are more susceptible to infection (Cardenas-Garcia et al. 2015), strict biosecurity protocols must ensure that there is an adequate period of time after the birds are vaccinated and before being exposed to infectious organisms. Key biosecurity practices include not only accurate record keeping but also to design and strictly follow proper vaccination programs and identify management practices that may facilitate the introduction of virulent NDV or practices that lead to the development of stressful conditions that will hinder an optimal immune response. Key record-keeping practices are necessary to assist in the early detection of disease and are crucial in preventing the spread of NDV into multiple locations. They include the recording of mortality, necropsies results, and proper carcass disposal. Carcass disposal is critical to control ND outbreaks because virulent NDV can remain viable in the tissue of infected birds for weeks and become a source of environmental contamination or direct infection of susceptible birds (Afonso and Miller 2014). Therefore, biosecurity practices that also prevent virulent NDV from contaminating the litter and water are crucial in preventing ND outbreaks (Afonso and Miller 2014). A common practice in Latin America is to use the litter from hens to fertilize agricultural fields, often situated next to poultry operations, without having it properly treated to prevent carrying infectious agents such as NDV.

Additional biosecurity practices used in farms involve the elimination of species that may become carriers of diseases such as pigeons, ducks, and other avian species. We have conducted a survey in the area of Jalisco, Mexico, from fall of 2014 to winter of 2015 in which 82 species of wild birds were identified to interact at some level with poultry houses. However, species of the family Icteridae comprised the most abundant group. A network-theory model provided a value that identified the highest ranked species to be the Mexican Great-tailed Grackle and the Barn Swallow; making those two species potential hosts for disease transmission of pathogens in the wild bird-poultry interface (Valdez-Gómez et al. 2017). Other factors such as restricted access for roads and visitors, cleaning and disinfection of vehicles, and resting periods between flocks are also positive factors in preventing infections.

Biosecurity programs also have the benefits of preventing the birds from being exposed to other live immunosuppressive agents (infectious bursal disease virus, chicken infectious anemia virus, Marek’s disease virus, fowl adenoviruses, etc.) (Hoerr 2010). Immunosuppressed birds are unable to mount a proper immune response to any vaccines; they are administered in the future and are more susceptible to NDV infections compared with birds that are vaccinated and not immunosuppressed (Perozo et al. 2008). Perozo et al. conducted a vaccine-challenge trial in commercial broilers reared in either the field or in an experimental isolated setting (Perozo et al. 2008). Their vaccination strategy included dual (live/killed) priming of 1-day-old chicks plus two live NDV and infectious bursal disease virus (IBDV) field vaccinations at days 7 and 17, followed by a challenge with the VEN-611 isolate at day 28. At 28 days post vaccination, field vaccinates displayed significantly lower NDV ELISA antibody titers than the experimentally reared birds. During the challenge, only 57.1% of field-vaccinated birds survived the lethal challenge, differing (*P* < 0.05) in comparison with 90.5% survival in the experimental farm (Perozo et al. 2008). After bursal integrity assessment at 14, 21, and 28 days of age, macroscopic lesions and the relative bursa/body weight ratio demonstrated that animals in the field suffered severe lymphoid depletion of the follicles, increased amount of stroma between follicles, and severe follicular atrophy (Perozo et al. 2008). ELISA results confirmed high levels of IBV antibodies suggesting that despite vaccination, the field birds developed IBD, compromising the ability of the birds to mount an adequate immune response to the NDV vaccination, and subsequent field challenge with virulent NDV endemic in the area (Perozo et al. 2008). The differences in protection observed in the field-vaccinated birds suggested that management, environmental, and/or immunosuppressive factors may be affecting ND control and vaccine efficacy in the country. Furthermore, they suggest the need to implement comprehensive diagnostics such as those based on random next-generation sequencing, some of which are currently available in research laboratories across the globe (Dimitrov et al. 2017a, b).

Stressful rearing situations, in addition to live agents, may contribute to immunosuppression (Hoerr 2010), and proper and humane environments are important factors necessary to prevent disease. Immunosuppression also affects the outcome when vaccinated birds are infected with virulent NDV. The atrophy and lesions found in the reproductive organs of layers is one outcome that may be attributed to the lack of complete neutralization of virulent NDV combined with physiological changes and other unknown factors. Virulent NDV is known to activate the hypothalamic-pituitary-adrenal (HPA) axis and the sympathetic-adrenal-medullary (SAM) system, which brings about changes in the concentration of glucocorticoids and catecholamines in plasma (Silverman et al. 2005). The main glucocorticoid secreted by the adrenal gland is corticosterone, a compound often associated with stress.

NDV is capable of increasing the concentration of corticosterone up to sixfold in the serum of infected birds (Park et al. 2007). The presence of corticosterone in birds impacts the oviduct preventing the release of pituitary hormones LH and FSH (luteinizing hormone and follicle stimulating hormone, respectively). These two hormones are responsible for stimulating the production of the steroid hormones that encourage the formation of the white and shell of the egg (Murphy et al. 1999; Downing and Bryden 2008; Ahmed et al. 2014; Bwala et al. 2011). Likewise, in breeding hens, the presence of corticosterone can be transferred to the egg and affect embryonic development, manifesting in negative phenotypic effects after hatching (Saino et al. 2005; Downing and Bryden 2008; Bwala et al. 2011).

### D. Vaccination programs of the future

Vaccination against NDV dates back more than 60 years (Dimitrov et al. 2017a, b). However, to date, the virus continues to cause outbreaks in numerous poultry farming areas in South America (Supplementary Table 2) and around the world. As mentioned earlier, antigenic differences (Fig. 2) between the vaccine strains belonging to genotypes I and II, and the virulent NDV strains of genotypes V, VI, VII, and XIII causing ND outbreaks may contribute to the increased secretion and maintenance of the virus in vaccinated flocks or the environment (Kapczynski and King 2005; Miller et al. 2013). Virulent strains from new genotypes continue to be isolated from chickens suggesting that vaccination using conventional strategies has not been, nor will be the complete solution to control the disease. The challenge is ever increasing, due to the diversity of viruses that appear every year; thus, it is expected that sooner or later adjustments to increase antigenic similarity between the vaccines and challenge viruses will be needed.

As depopulation is often not a viable economical alternative and biosecurity is limited to highly advanced farms, it has been suggested that other strategies need to be developed to enable better control of ND. These strategies should not only aim to prevent mortality but also to reduce the quantity of challenge virus particles excreted, thus reducing the persistence of the virus in the flock, with both parameters being indicators of vaccine efficacy (Miller et al. 2010). For that purpose, one strategy proposed in recent years has been the development of antigenically matched vaccines; i.e., vaccines formulated based on a vaccine viral seed that belongs to the same genotype as the challenge virus. This strategy has shown to be effective for both inactive vaccines and live vaccines developed from homologous genotypes of the challenge virus, to increase efficacy against virulent challenge strains circulating in the field, and above all, on reducing the number of excreted viral particles (Miller et al. 2007; 2009a; Hu et al. 2009; Absalón et al. 2012b; Cardenas-Garcia et al. 2015; Dimitrov et al. 2017a, b).

Antigenically matched low virulence vaccine seeds are normally created through reverse genetics following established procedures (Cardenas-Garcia and Afonso 2017; Molouki and Peeters 2017). As of 2018, two strategies have been described to obtain homologous low virulence vaccine seed from virulent viruses. In the first strategy, the vaccine seed consists of a recombinant NDV in which modifications were made to the nucleotides encoding the basic amino acids of the F protein cleavage site. These viruses maintain the genetic characteristics of the original NDV, with the exception of the amino acids that determine virulence (Hu et al. 2009, 2011). A second strategy consists of using the genome or “backbone” of an NDV strain (LaSota, for example) and replacing the genes that totally or partially encode for the antigenic proteins F and HN of virulent strains. As in the first strategy, these viruses also involve modifications at the cleavage site to give the resulting recombinant NDV with a phenotype of low virulence (Absalón et al. 2012b; Cardenas-Garcia et al. 2015).

Antigenically matched NDV vaccines have demonstrated to be efficient for preventing mortality while significantly reducing viral excretion (Hu et al. 2009, 2011; Absalón et al. 2012b; Cardenas-Garcia et al. 2015). It has been reported that immunization with recombinant NDV vaccines of genotype VII conferred protection against mortality with the absence of clinical signs when challenged with virulent NDV strains of genotype VII similar to those circulating in Asia (Ji et al. 2018). Similarly, a recombinant NDV capable of expressing the antigenic proteins F and HN of a virulent NDV (A/chicken/Mexico/P05/2005; short name NDV-P05) of genotype V, enzootic in Mexico has been developed. Experimental studies in birds immunized with this product have shown greater protection against the homologous antigen. This protection was observed in the reduction in viral excretion of the challenge virus via trachea and cloaca compared to the LaSota strain (Absalón et al. 2012b); however, when large amounts of challenge virus are used, and under-vaccination conditions that mimic field conditions, statistically significant differences in survival were observed (Cardenas-Garcia et al. 2015). The strategy of using a recombinant HVT expressing the F protein should be applicable to obtain vaccines that are genetically matched to virulent field viruses circulating in Latin America in order to produce more specific antibodies.

## Epidemiological similarities to other ND endemic countries

Latin America’s poultry production industry shares commonalities with other countries across the world that struggle with the repeated occurrence of ND. In these countries, where ND is endemic, there are epidemiological factors, production systems, and socioeconomic and cultural practices that allow for the maintenance of virus and thus, the disease (Wajid et al. 2017). The most common similarities are the commercial vaccines administered and the vaccination practices employed, e.g., route and frequency of administration. Most countries utilize the same type of live and/or inactivated vaccines based on older genotypes that most often do not achieve a significant reduction of virus replication and shedding of virulent NDV from vaccinated birds when they are infected in the field (Rehmani et al. 2015). Under optimal conditions, these live NDV vaccines can achieve a 2 or 3 log reduction in virus shedding. However, this is less likely when early challenges from virulent NDV present in the environment and insufficient biosecurity practices occur. The continuous evolution of the existing NDV genotypes and reoccurring outbreaks suggest that the ND vaccines alone have not achieved the goal of preventing virus circulation.

Another commonality of all countries with endemic NDV is the existence of large production facilities concentrated in small geographic regions, thus creating a very high density of poultry farms in close proximity to backyard flocks (Wajid et al. 2017). The high density is likely to facilitate transmission through air and water, as well as through vehicular movement and shared equipment, supplies, and personnel. A third shared feature is the lack of efficient stamping-out procedures or enforcement of sanitary and containment regulations. In most of Latin America, as well as many countries in Asia and Africa, there are no effective systems for government compensation to poultry producers when a quarantine or an eradication measure is needed. It is also possible that some smallholders may have no awareness that the disease should be reported. Either of these deficiencies results in the underreporting of new cases to authorities. Furthermore, to avoid losses when mortalities occur, it is not uncommon to quickly sell the remaining stocks of apparently healthy birds to slaughterhouses and markets, thus contributing to further spread of the virus to new geographic locations.

The fourth similarity is the use of untreated poultry manure in agricultural applications. Manure from broilers and hens is sold to feed cattle or used as fertilizers in agricultural fields. As the virus is relatively stable during the cold or wet seasons and can survive up to several months in litter, it is likely to be infectious while being transported or during the dispersal of manure onto agricultural fields (Voss-Rech et al. 2017). In most endemic countries, there are no standard operating procedures for composting, nor is there a systematic evaluation of composting outcomes or virus inactivation. Consequently, without any government control, large poultry facilities normally sell huge volumes of infected bedding material (litter) without any restriction, inadvertently increasing the environmental load of NDV.

A fifth common characteristic is the lack of regular active surveillance programs. While some countries have incorporated active surveillance because of continuous avian influenza outbreaks, for most countries, there is a lack of sufficient funds or capacities for effective wide-scale surveillance efforts to cover all production facilities and regions (Moila et al. 2017). Diagnostics could be expensive in some areas, and certain producers, particularly the smaller farms, cannot afford to monitor the presence of virulent NDV, complicated with the universal use of live NDV vaccines. Another global practice that is common, but poorly documented, is the production of autologous vaccines by small local laboratories. Small laboratories often have neither the resources to detect mixed infections with multiple organisms in the cultures they are growing nor the ability to ensure the proper safety testing of inactivated vaccines.

Finally, employing proper biosecurity protocols is very complex, often requires massive investment in infrastructure and management practices, and is even difficult for the most well-funded companies in countries where NDV is not endemic (Wajid et al. 2017). At a minimum, biosecurity practices designed to prevent contact with backyard poultry have been introduced in some countries where ND is endemic. In Mexico, for example, poultry producers provide chickens or vaccinated hens to employees for their own consumption, thus, limiting the number of birds raised by poultry workers in their own backyards. In Peru and other countries in Latin America, vaccine companies and the government subsidize vaccination programs for backyard poultry owners located around the production facilities. Despite massive efforts and funds spent, ND has not been eradicated anywhere solely utilizing vaccination and biosecurity.

Eradication can only be addressed with a good understanding of the epidemiology of the disease, the likely sources of re-introduction, and the best vaccination and biosecurity programs for each farm, along with the use of culling, all combined. Unfortunately, the cost of eradication is normally far greater than the cost of controlling the disease, and experience demonstrates that substantial financial support, not only during the eradication campaign but also afterward for continued active surveillance, is required. However, the technology for eradication is available, and the practicality of eradication has been demonstrated (for example, in the recent introduction of Asian H5 avian influenza viruses in the USA).

## Conclusions

Prevention and control strategies for Newcastle disease in endemic countries need improvement. Ideally, these would include measures that include culling, active surveillance, improved biosecurity, and stringent vaccination programs. Any strategy needs to be accompanied by measures that ensure strict compliance of policies and procedures, which would include adequate record keeping and control of movement and disposal of infected animals. As with the outbreaks in the USA (California 2002 and 2018), the measures might include control of the viruses replicating in backyard and pet species, together with control of viruses in commercial birds. The current reliance on the use of vaccines formulated from seed strains from older genotypes that are less antigenically similar to the virulent challenge viruses may not eliminate the continuing persistence of the virus in farms due to viral excretion from the vaccinated-but-infected birds or prevent egg drops in vaccinated layers (Pandarangga et al. 2016; Rehmani et al. 2015; Dimitrov et al. 2017a, b). The use of vaccines formulated with NDV strains belonging to the same genotype as the challenge NDV will likely induce more antigenically related antibodies than the commercial NDV vaccines available currently (Cardenas-Garcia et al. 2015) and hopefully will reduce opportunistic challenges in poorly vaccinated animals.

## Electronic supplementary material

ESM 1 (DOCX 69 kb)

## References

[CR1] Absalón AE, Mariano-Matias A, Vasquez-Marquez A, Morales-Garzon A, Cortes-Espinosa DV, Ortega-Garcia R, Lucio-Decanini E (2012). Complete genome sequence of a velogenic Newcastle disease virus isolated in Mexico. Virus Genes.

[CR2] Absalón, A.E., Cortés-Espinosa, D.V., Lucio-Decanini, E., Morales-Garzón, A., 2012b. Newcastle disease virus and the use thereof as a vaccine. Patent Request. WO 2012067483

[CR3] Absalón AE, Mariano-Matías A, García LJ, Morales-Garzón A, Toscano-Contreras A, Lucio-Decanini E, Cortés-Espinosa DV (2014). Complete genome analysis of velogenic Newcastle disease virus reference strain "Chimalhuacan": evolution of viral lineages in Mexico. Virus Genes.

[CR4] Afonso CL (2008). Not so fast on recombination analysis of Newcastle disease virus. Journal of Virology. Sept.

[CR5] Afonso CL, Miller PJ, Liu D (2014). Newcastle disease virus. Security sensitive microbes and toxins.

[CR6] Afonso CL, Amarasinghe GK, Banyai K, Bao Y, Basler CF, Bavari S, Bejerman N, Blasdell KR, Briand FX, Briese T, Bukreyev A, Calisher CH, Chandran K, Chéng J, Clawson AN, Collins PL, Dietzgen RG, Dolnik O, Domier LL, Dürrwald R, Dye JM, Easton H, Farkas SL, Freitas-Astúa J, Formenty P, Fouchier RA, Fú Y, Ghedin E, Goodin MM, Hewson R, Horie M, Hyndman TH, Jiāng D, Kitajima EW, Kobinger GP, Kondo H, Kurath G, Lamb RA, Lenardon S, Leroy EM, Li CX, Lin XD, Liú L, Longdon B, Marton S, Maisner A, Mühlberger E, Netesov SV, Nowotny N, Patterson JL, Payne SL, Paweska JT, Randall RE, Rima BK, Rota P, Rubbenstroth D, Schwemmla M, Shi M, Smithers SJ, Stenglein MD, Stone DM, Takada A, Terregino C, Tesh RB, Tian JH, Tomonaga K, Tordo N, Towner JS, Vasilakis N, Verbeek M, Volchkov VE, Wahl-Jensen V, Walsh JA, Walker PJ, Wang D, Wang LF, Wetzel T, Whitfield AE, Xiè JT, Yuen KY, Zhang YZ, Kuhn JH (2016). Taxonomy of the order Mononegavirales: update 2016. Archives of Virology.

[CR7] Ahmed AA, Ma W, Ni Y, Zhou Q, Zhao R (2014). Embryonic exposure to corticosterone modifies aggressive behavior through alterations of the hypothalamic pituitary adrenal axis and the serotonergic system in the chicken. Hormone Behavior.

[CR8] Aldous E, Alexander D (2001). Detection and differentiation of Newcastle disease virus (avian paramyxovirus type 1). Avian Pathology.

[CR9] Aldous EW, Mynn JK, Banks J, Alexander DJ (2003). A molecular epidemiological study of avian paramyxovirus type 1 (Newcastle disease virus) isolates by phylogenetic analysis of a partial nucleotide sequence of the fusion protein gene. Avian Pathology.

[CR10] Alexander, D.J., Bell, J.G., Alders, R.G., 2004. A technology review, Newcastle disease with special emphasis on its effect on village chickens. FAO Animal production and health book No. 161. Food andAgriculture Organization of United Nations, Rome, 23-63.

[CR11] Amarasinghe GK, Bào Y, Basler CF, Bavari S, Beer M, Bejerman N, Blasdell KR, Bochnowski A, Briese T, Bukreyev A, Calisher CH, Chandran K, Collins PL, Dietzgen RG, Dolnik O, Dürrwald R, Dye JM, Easton AJ, Ebihara H, Fang Q, Formenty P, Fouchier RAM, Ghedin E, Harding RM, Hewson R, Higgins CM, Hong J, Horie M, James AP, Jiāng D, Kobinger GP, Kondo H, Kurath G, Lamb RA, Lee B, Leroy EM, Li M, Maisner A, Mühlberger E, Netesov SV, Nowotny N, Patterson JL, Payne SL, Paweska JT, Pearson MN, Randall RE, Revill PA, Rima BK, Rota P, Rubbenstroth D, Schwemmle M, Smither SJ, Song Q, Stone DM, Takada A, Terregino C, Tesh RB, Tomonaga K, Tordo N, Towner JS, Vasilakis N, Volchkov VE, Wahl-Jensen V, Walker PJ, Wang B, Wang D, Wang F, Wang LF, Werren JH, Whitfield AE, Yan Z, Ye G, Kuhn JH (2017). Taxonomy of the order Mononegavirales: update 2017. Archives of Virology.

[CR12] Amarasinghe, G.K., Aréchiga Ceballos, N.G., Banyard, A.C., Basler, C.F., Bavari, S., Bennett, A.J., Blasdell, K.R., Briese, T., Bukreyev, A., Cai, Y., Calisher, C.H., Campos Lawson, C., Chandran, K., Chapman, C.A., Chiu, C.Y., Choi, K.S., Collins, P.L., Dietzgen, R.G., Dolja, V.V., Dolnik, O., Domier, L.L., Dürrwald, R., Dye, J.M., Easton, A.J., Ebihara, H., Echevarría, J.E., Fooks, A.R., Formenty, P.B.H., Fouchier, R.A.M., Freuling, C.M., Ghedin, E., Goldberg, T.L., Hewson, R., Horie, M., Hyndman, T.H., Jiāng, D., Kityo, R., Kobinger, G.P., Kondō, H., Koonin, E.V., Krupovic, M., Kurath, G., Lamb, R.A., Lee, B., Leroy, E.M., Maes, P., Maisner, A., Marston, D.A., Mor, S.K., Müller, T., Mühlberger, E., Ramírez, V.M.N., Netesov, S.V., Ng, T.F.F., Nowotny, N., Palacios, G., Patterson, J.L., Paweska, J.T., Payne, S.L., Prieto, K., Rima, B.K., Rota, P., Rubbenstroth, D., Schwemmle, M., Siddell, S., Smither, S.J., Son, Q., Song, T., Stenglein, M.D., Stone, D.M., Takada, A., Tesh, R.B., Thomazelli, L.M., Tomonaga, K., Tordo N., Towner, J.S., Vasilakis, N., Vázquez-Morón, S., Verdugo, C., Volchkov, V.E., Wahl, V., Walker, P.J., Wang, D., Wang, L.F., Wellehan, J.F.X., Wiley, M.R., Whitfield, A.E., Wolf, Y.I., Yè, G., Zhāng, Y.Z., Kuhn, J.H. 2018. Taxonomy of the order Mononegavirales: update 2018. Archives of Virology 163, 2283-2294. 10.1007/s00705-018-3814-x 10.1007/s00705-018-3814-x PMC6076851 29637429

[CR13] Ayala AJ, Dimitrov KM, Becker CR, Goraichuk IV, Arns CW, Bolotin VI, Ferreira HL, Gerilovych AP, Goujgoulova GV, Martini MC, Muzyka DV, Orsi MA, Scagion GP, Silva RK, Solodiankin OS, Stegniy BT, Miller PJ, Afonso CL (2016). Presence of vaccine-derived Newcastle disease viruses in wild birds. PLoS One.

[CR14] Bell, J.G., 2001. A comparison of the different vaccine available for the control of Newcastle disease in village chickens. In: Alders, R.G. and Spradbrow, P.B. ed., PR103, SADC Planning Workshop on Newcastledisease control in village chickens. Proceedings of an International Workshop, Maputo, Mozambique, 6-9March, 2000. ACIAR Proceedings No. 103, 56–60. https://www.aciar.gov.au/node/7876

[CR15] Berhane Y, Hisanaga T, Xu W, Mosos Campos NA, Kehler H, Calderón Parra CP, Pasick J (2017). Characterization of Colombian serotype 1 avian paramyxoviruses, 2008-2010. Virus Genes.

[CR16] Bertran K, Susta L, Miller PJ, Hester P (2017). Avian influenza virus and Newcastle disease virus. Egg innovation and strategies for improvement.

[CR17] Brown, J.A., Gongora, V., Hartley, D., Oura, C., 2018. A review of eight high-priority, economically important viral pathogens of poultry within the Caribbean region. Veterinary Science. 5, 14. 10.3390/vetsci5010014 10.3390/vetsci5010014 PMC5876562 29373488

[CR18] Bwala DG, Fasina FO, Van Wyk A, Duncan NM (2011). Effects of vaccination with lentogenic vaccine and challenge with virulent Newcastle disease virus (NDV) on egg production in commercial and SPF chickens. International Journal Poultry Science.

[CR19] Cardenas Garcia S, Navarro Lopez R, Morales R, Olvera MA, Marquez MA, Merino R, Miller PJ, Afonso CL (2013). Molecular epidemiology of Newcastle disease in Mexico and the potential spillover of viruses from poultry into wild bird species. Applied and Environmental Microbiology.

[CR20] Cardenas-Garcia S, Afonso CL (2017). Reverse genetics of Newcastle disease virus. Methods Molecular Biology.

[CR21] Cardenas-Garcia S, Diel DG, Susta L, Lucio-Decanini E, Yu Q, Brown CC, Miller PJ, Afonso CL (2015). Development of an improved vaccine evaluation protocol to compare the efficacy of Newcastle disease vaccines. Biologicals.

[CR22] Chumbe A, Izquierdo-Lara R, Tataje L, Gonzalez R, Cribillero G, González AE, Fernández-Diaz M, Icochea E (2017). Pathotyping and phylogenetic characterization of Newcastle disease viruses isolated in Peru: defining two novel subgenotypes within genotype XII. Avian Diseases.

[CR23] Cornax I, Miller PJ, Afonso CL (2012). Characterization of live LaSota vaccine strain-induced protection in chickens upon early challenge with a virulent Newcastle disease virus of heterologous genotype. Avian Diseases.

[CR24] Courtney SC, Susta L, Gomez D, Hines NL, Pedersen JC, Brown CC, Miller PJ, Afonso CL (2013). Highly divergent virulent isolates of Newcastle disease virus from the Dominican Republic are members of a new genotype that may have evolved unnoticed for over 2 decades. Journal of Clinical Microbiology.

[CR25] Diel DG, da Silva DH, Liu H, Wang Z, Miller PJ, Afonso CL (2012). Genetic diversity of avian paramyxovirus type 1: proposal for a unified nomenclature and classification system of Newcastle disease virus genotypes. Infection Genetics and Evolution.

[CR26] Diel DG, Miller PJ, Wolf PC, Mickley RM, Musante AR, Emanueli DC, Shively KJ, Pedersen K, Afonso CL (2012). Characterization of Newcastle disease viruses isolated from cormorant and gull species in the United States in 2010. Avian Diseases..

[CR27] Diel DG, Susta L, Cardenas Garcia S, Killian ML, Brown CC, Miller PJ, Afonso CL (2012). Complete genome and clinicopathological characterization of a virulent Newcastle disease virus isolated from poultry in South America. Journal of Clinical Microbiology.

[CR28] Dimitrov KM, Bolotin V, Muzyka D, Goraichuk IV, Solodiankin O, Gerilovych A, Stegniy B, Goujgoulova GV, Silko NY, Pantin-Jackwood MJ, Miller PJ, Afonso CL (2016). Repeated isolation of virulent Newcastle disease viruses of sub-genotype VIId from backyard chickens in Bulgaria and Ukraine between 2002 and 2013. Archives of Virology.

[CR29] Dimitrov KM, Lee DH, Williams-Coplin D, Olivier TL, Miller PJ, Afonso CL (2016). Newcastle disease viruses causing recent outbreaks worldwide show unexpectedly high genetic similarity to historical virulent isolates from the 1940s. Journal of Clinical Microbiology.

[CR30] Dimitrov KM, Ramey AM, Qui X, Bahl J, Afonso CL (2016). Temporal, geographic, and host distribution of avian paramyxovirus 1 (Newcastle disease virus). Infection Genetics and Evolution.

[CR31] Dimitrov KM, Afonso CL, Miller PJ (2017). Newcastle disease vaccines—a solved problem or a continuous challenge?. Veterinary Microbiology.

[CR32] Dimitrov KM, Sharma P, Volkening JD, Goraichuk IV, Wajid A, Rehmani SF, Basharat A, Shittu I, Joannis TM, Miller PJ, Afonso CL (2017). A robust and cost-effective approach to sequence and analyze complete genomes of small RNA viruses. Virology Journal.

[CR33] Dortmans, J.C., Koch, G., Rottier, P.J., Peeters, B.P., 2011. Virulence of Newcastle disease virus: what is known so far?. Veterinary Research 42, 122. 10.1186/1297-9716-42-122 10.1186/1297-9716-42-122 PMC3269386 22195547

[CR34] Downing JA, Bryden WL (2008). Determination of corticosterone concentrations in egg albumen: a non-invasive indicator of stress in laying hens. Physiology and Behavior.

[CR35] Erdei J, Erdei J, Bachir K, Kaleta EF, Shortridge KF, Lomniczi B (1987). Newcastle disease vaccine (LaSota) strain specific monoclonal antibody. Archives of Virology.

[CR36] Fernandes CC, Varani AM, Lemos EG, de Miranda VF, Silva KR, Fernandos FS, Montassier MF, Montassier HJ (2014). Molecular and phylogenetic characterization based on the complete genome of a virulent pathotypes of Newcastle disease virus isolated in the 1970s in Brazil. Infection Genetics and Evolution.

[CR37] Han GZ, He CQ, Ding NZ, Ma LY (2008). Identification of a natural multi-recombinant of Newcastle disease virus. Virology..

[CR38] He Y, Taylor TL, Dimitrov KM, Butt SL, Stanton JB, Goraichuck IV, Fenton H, Poulson R, Zhang J, Brown CC, Ip HS, Isodoro-Ayza M, Afonso CL (2018). Whole-genome sequencing of genotype VI Newcastle disease viruses from formalin-fixed paraffin-embedded tissues from wild pigeons reveals continuous evolution and previously unrecognized genetic diversity in the U.S. Virology Journal.

[CR39] Hoerr FJ (2010). Clinical aspects of immunosuppression in poultry. Avian Diseases.

[CR40] Hu S, Ma H, Wu Y, Liu W, Wang X, Liu Y, Liu X (2009). A vaccine candidate of attenuated genotype VII Newcastle disease virus generated by reverse genetics. Vaccine.

[CR41] Hu Z, Hu S, Meng C, Wang X, Zhu J, Liu X (2011). Generation of a genotype VII Newcastle disease virus vaccine candidate with high yield in embryonated chicken eggs. Avian Diseases.

[CR42] Igwe AO, Afonso CL, Ezema WS, Brown CC, Okoye JOA (2018). Pathology and distribution of velogenic viscerotropic Newcastle disease virus in the reproductive system of vaccinated and unvaccinated laying hens (*Gallus gallus domesticus*) by immunohistochemical labeling. Journal of Comparative Pathology.

[CR43] Iorio RM, Bratt MA (1984). Monoclonal antibodies as functional probes of the HN glycoprotein of Newcastle disease virus: antigenic separation of the hemagglutinating and neuraminidase sites. Journal of Immunology.

[CR44] Ji Y, Liu T, Cui X, Yu Q, Wang Z, Zhang J, Li Y, Zhu Q (2018). A novel genotype VII Newcastle disease virus vaccine candidate generated by mutation in the L and F genes confers improved protection in chickens. Veterinary Microbiology.

[CR45] Kaleta EF, Baldauf C, Alexander DJ (1988). Newcastle disease in free-living and pet birds. Newcastle disease.

[CR46] Kapczynski DR, King DJ (2005). Protection of chickens against overt clinical disease and determination of viral shedding following vaccination with commercially available Newcastle disease virus vaccines upon challenge with highly virulent virus from the California 2002 exotic Newcastle disease outbreak. Vaccine.

[CR47] Lee CW, Senne DA, Suarez DL (2004). Effect of vaccine use in the evolution of Mexican lineage H5N2 avian influenza virus. Journal of Virology.

[CR48] Liu XF, Wan HQ, Ni XX, Wu YT, Liu WB (2003). Pathotypical and genotypical characterization of strains of Newcastle disease virus isolated from outbreaks in chicken and goose flocks in some regions of China during 1985-2001. Archives of Virology.

[CR49] Mayo MA (2002). A summary of taxonomic changes recently approved by ICTV. Archives of Virology.

[CR50] McFerran JB, McCracken RM, Alexander DJ (1988). Newcastle disease. Newcastle disease.

[CR51] Merino R, Villegas H, Quintana JA, Calderon N (2009). Characterization of Newcastle disease viruses isolated from chicken, gamefowl, pigeon and quail in Mexico. Veterinary Research Communications.

[CR52] Miller PJ, Koch G, Swayne DE, Glisson JR, McDougald LR, Nolan LK, Suarez DL, Nair V (2013). Newcastle disease. Diseases of Poultry.

[CR53] Miller PJ, King DJ, Afonso CL, Suarez DL (2007). Antigenic differences among Newcastle disease virus strains of different genotypes used in vaccine formulation affect viral shedding after a virulent challenge. Vaccine..

[CR54] Miller PJ, Estevez C, Yu Q, Suarez DL, King DJ (2009). Comparison of viral shedding following vaccination with inactivated and live Newcastle disease vaccines formulated with wild-type and recombinant viruses. Avian Diseases.

[CR55] Miller PJ, Kim LM, Ip HS, Afonso CL (2009). Evolutionary dynamics of Newcastle disease virus. Virology.

[CR56] Miller PJ, Decanini EL, Afonso CL (2010). Newcastle disease: evolution of genotypes and the related diagnostic challenges. Infection Genetics and Evolution.

[CR57] Miller PJ, Afonso CL, El Attrache J, Dorsey KM, Dorsey SC, Guo Z, Kapczynski DR (2013). Effects of Newcastle disease virus vaccine antibodies on the shedding and transmission of challenge virus. Developmental Comparative Immunology.

[CR58] Moila, S., Grosbois, V., Kamissoko, B., Sidibe, M.S., Sissoko, K.D., Traore, I., Diakite, A., Pfeiffer, D.U., 2017. Longitudinal study of avian influenza and Newcastle disease in village poultry, Mali, 2009-2011. Avian Diseases. 61, 165-177. 10.1637/11502-092616-Reg.1 10.1637/11502-092616-Reg.1 28665735

[CR59] Molouki A, Peeters B (2017). Rescue of recombinant Newcastle disease virus: current cloning strategies and RNA polymerase provision systems. Archives of Virology.

[CR60] Mumma JO, Thaxton J, Vizzier-Thaxton Y, Dodson W (2006). Physiological stress in laying hens. Poultry Science.

[CR61] Murphy, F.A., Gibbs, E.P.J., Horzinek, M.C., Studdert, M.J., 1999. Paramyxoviridae. In: Veterinary Virology, 3rd edition. Chapter 26, Academic Press, San Diego, CA. 405–458

[CR62] Nayak B, Dias FM, Kumar S, Paldurai A, Collins PL, Samal SK (2012). Avian paramyxovirus serotypes 2-9 (APMV-2-9) vary in the ability to induce protective immunity in chickens against challenge with virulent Newcastle disease virus (APMV-1). Vaccine.

[CR63] OIE (2006). World Animal Health Information Database (WAHIS Interface)-Version 1. World Organisation for Animal Health (OIE). Newcastle disease virus. Jan 2006 -Dec 2006, Brasil. https://www.oie.int/wahis_2/public/wahid.php/Diseaseinformation/statusdetail. Accessed 7 March 2019

[CR64] Palya V, Kiss I, Tatar-Kis T, Mato T, Felfoldi B, Gardin Y (2012). Advancement in vaccination against Newcastle disease: recombinant HVT NDV provides high clinical protection and reduces challenge virus shedding with the absence of vaccine reactions. Avian Diseases.

[CR65] Pandarangga P, Brown CC, Miller PJ, Haddas R, Rehmani SF, Afonso CL, Susta L (2016). Pathogenesis of new strains of Newcastle disease virus from Israel and Pakistan. Veterinary Pathology.

[CR66] Park J, Kim M, Na G, Jeon I, Kwon YK, Kim JH, Youn H, Koo Y (2007). Glucocorticoids modulate NF-κB-dependent gene expression by up-regulating FKBP51 expression in Newcastle disease virus-infected chikens. Journal of Molecular and Cellular Endocrinology.

[CR67] Pedersen JC, Senne DA, Woolcock PR, Kinde H, King DJ, Wise MG, Panigrahy B, Seal BS (2004). Phylogenetic relationship among virulent Newcastle disease virus isolates from the 2002-2003 outbreak in California and other recent outbreaks in North America. Journal of Clinical Microbiology.

[CR68] Perozo F, Merino R, Afonso CL, Villegas P, Calderon N (2008). Biological and phylogenetic characterization of virulent Newcastle disease virus circulating in Mexico. Avian Diseases.

[CR69] Perozo F, Marcano R, Afonso CL (2012). Biological and phylogenetic characterization of genotype VII Newcastle disease virus from Venezuela: efficacy of field vaccination. Journal of Clinical Microbiology.

[CR70] Rao MS, Raj GD, Manohar BM (2002). An in vitro and in vivo evaluation of the virulence of Newcastle disease virus and vaccines for the chicken reproductive tract. Avian Pathology.

[CR71] Rauw F, Gardin Y, Palya V, Anbari S, Lemaire S, Boschmans M, van den Berg T, Lambrecht B (2010). Improved vaccination against Newcastle disease by an in ovo recombinant HVT-ND combined with an adjuvanted live vaccine at day-old. Vaccine.

[CR72] Rehmani SF, Wajid A, Bibi T, Nazir B, Mukhtar N, Hussain A, Lone NA, Yaqub T, Afonso CL (2015). Presence of virulent Newcastle disease virus in vaccinated chickens in farms in Pakistan. Journal of Clinical Microbiology.

[CR73] Rue CA, Susta L, Cornax I, Brown CC, Kapczynski DR, Suarez DL, King DJ, Miller PJ, Afonso CL (2011). Virulent Newcastle disease virus elicits a strong innate immune response in chickens. Journal of General Virology.

[CR74] Sá E, Silva M, Susta L, Moresco K, Swayne DE (2016). Vaccination of chickens decreased Newcastle disease virus contamination in eggs. Avian Pathology.

[CR75] Sabra M, Dimitrov KM, Goraichuk IV, Wajid A, Sharma P, Williams-Coplin D, Basharat A, Rehmani SF, Muzyka DV, Miller PJ, Afonso CL (2017). Phylogenetic assessment reveals continuous evolution and circulation of pigeon-derived virulent avian avulaviruses 1 in Eastern Europe, Asia, and Africa. BMC Vet. Res.

[CR76] Saino N, Romano M, Ferrari RP, Martinelli R, Moller AP (2005). Stressed mothers lay eggs with high corticosterone levels which produce low-quality offspring. Journal of Experimental Zoology Part A Comparative Experimental Biology.

[CR77] Santos JA, Silva RA, Brada W, Marinho E, Cunha RG (1954). A ocorrência da doença de Newcastle no Brasil. (Nota Previa). Reviews of Production Animals, (Rio).

[CR78] Silverman MN, Pearce BD, Biron CA, Miller AH (2005). Immune modulation of the hypothalamic-pituitary-adrenal (HPA) axis during viral infection. Viral Immunology.

[CR79] Snoeck CJ, Owoade AA, Couacy-Hymann E, Alkali BR, Okwen MP, Adeyanju AT, Komoyo GF, Nakouné E, Le Faou A, Muller CP (2013). High genetic diversity of Newcastle disease virus in poultry in West and Central Africa: cocirculation of genotype XIV and newly defined genotypes XVII and XVIII. Journal of Clinical Microbiology.

[CR80] Sondermeijer PJ, Claessens JA, Jenniskens PE, Mockett AP, Thijssen RA, Willemse MJ, Morgan RW (1993). Avian herpesvirus as a live viral vector for the expression of heterologous antigens. Vaccine.

[CR81] Song Q, Cao Y, Li Q, Gu M, Zhong L, Hu S, Wan H, Liu X (2011). Artificial recombination may influence the evolutionary analysis of Newcastle disease virus. Journal of Virology.

[CR82] Souza, S.O., Fedo, G., Dupont, P.M., Leite-Filho, R.V., Teifke, J.P., Pavarini, S.P., Canal, C.W, Driemeier, D. 2018. Pathological and molecular findings of avian avulavirus type 1 outbreak in pigeons (Columba livia) of southern Brazil. Brazilian Veterinary Research. abstract in English. 38, 12. 10.1590/1678-5150-pvb-5528

[CR83] Sun HL, Wang YF, Tong GZ, Zhang PJ, Miao DY, Zhi HD, Wang M, Wang M (2008). Protection of chickens from Newcastle disease and infectious laryngotracheitis with a recombinant fowlpox virus co-expressing the FHN genes of Newcastle disease virus and gB gene of infectious laryngotracheitis virus. Avian Diseases..

[CR84] Susta L, Hamal KR, Miller PJ, Cardenas-Garcia S, Brown CC, Pedersen JC, Gongora V, Afonso CL (2014). Separate evolution of virulent Newcastle disease viruses from Mexico and Central America. Journal of Clinical Microbiology.

[CR85] Taylor TL, Miller PJ, Olivier TL, Montiel E, Cardenas Garcia S, Dimitrov KM, Williams-Coplin D, Afonso CL (2017). Repeated challenge with virulent Newcastle disease virus does not decrease efficacy of vaccines. Avian Diseases.

[CR86] Valdez-Gómez, H.E., Navarro-López, R., Vázquez-Mendoza, L.F., Zalapa-Hernández, M., Guerroro-Hernández, I., Fonseca-Delgado, V., Márquez-Ruiz, M.A., Afonso, C.L. 2017. Risk factors for the transmission of infectious diseases agents at the wild birds-commercial birds interface. A pilot study in the regio of the Altos De Jalisco, Mexico. Bulletin De l’Academie Veterinaire De France 170, 142–150. 10.4267/2042/62332

[CR87] van Boven M, Bouma A, Fabri TH, Katsma E, Hartog L, Koch G (2008). Herd immunity to Newcastle disease virus in poultry by vaccination. Avian Pathology.

[CR88] Voss-Rech, D., Trevisol, I.M., Brentano, L., Silva V.S., Rebelatto, R., Jaenisch, F.R.F., Okino, C.H., Mores, M.A.Z., Coldebella, A., Botton, S.A., Vaz, C.S.L. 2017. Impact of treatments for recycled broiler litter on the viability and infectivity of microoganisms. Veterinary Microbiology. 203. 308–314. 10.1016/j.vetmic.2017.03.020 10.1016/j.vetmic.2017.03.020 28619162

[CR89] Wajid A, Dimitrov KM, Wasim M, Rehmani SF, Basharat A, Bibi T, Arif S, Yaqub T, Tayyab M, Ababneh M, Sharma P, Miller PJ, Afonso CL (2017). Repeated isolation of virulent Newcastle disease viruses in poultry and captive non-poultry avian species in Pakistan from 2011 to 2016. Preventative Veterinary Medicine.

[CR90] Wise MG, Suarez DL, Seal BS, Pedersen JC, Senne DA, King DJ, Kapczynski DR, Spackman E (2004). Development of a real-time reverse-transcription PCR for detection of Newcastle disease virus RNA in clinical samples. Journal of Clinical Microbiology.

[CR91] Wiseman A, Berman EM (2017). Herd immunity to Newcastle disease virus in broiler flocks in Israel. Avian Pathology.

[CR92] Zhang R, Wang X, Su J, Zhao J, Zhang G (2010). Isolation and analysis of two naturally-occurring multi-recombination Newcastle disease viruses in China. Virus Research.

