# Supplementary material for: Epidemiology, control, and prevention of Newcastle disease in endemic regions: Latin America
Source: Trop Anim Health Prod. 2019 Mar 15;51(5):1033–48. doi: 10.1007/s11250-019-01843-z (PMC6520322; doi:10.1007/s11250-019-01843-z)
Supplement: Supplementary file 1 — (DOCX 69 kb) [file 11250_2019_1843_MOESM1_ESM.docx]

Table 1. NDV vaccines

|  | Vaccine name | Strain used | Company | Presentation | Usage | Vaccination |
| --- | --- | --- | --- | --- | --- | --- |
| 2 | Genovax 5 | NDV P05 | SANFER IASA | Live vaccine Freeze-dried | Resuspend in 30ml (ocular o nasal) or 25 ml sterile diluent for spray. | Birds of 1 day of age henceforth |
| 3 | Emulmax N5 | NDV P05 | SANFER IASA | Inactive vaccine (in oil) | Application 0.5 ml per bird for subcutaneous route | Age of application dependent on local farm practices |
| 4 | Emulmax IBH (viral inclusion body hepatitis +ND | NDV P05 and Fowl Adenovirus serotype 4 strains SHP-IAP/94 and SHP-IAP/95 | SANFER IASA | Inactive vaccine (in oil) | Administer 0.5 ml per bird for subcutaneous route | Administer in birds of 10 and 14 days of age |
| 6 | Emulmax ND+EDS+IB | NDV P05, Infectious Bronchitis virus (Massachusetts), and DAdV-1 (Duck Adenovirus strain PM2804) | SANFER IASA | Inactive vaccine (in oil) | Administer 0.5 ml per bird via subcutaneous route in the back of the neck | Laying hens of 12 to 14 weeks and breeders of 14 to 18 weeks of age, previous immunization done with a minimum of two applications of NDV live and IBV. |
| 7 | Emulmax ND+IB | NDV P05 and Infectious Bronchitis virus (Massachusetts) | SANFER IASA | Inactive vaccine (in oil) | Administer 0.5 ml vaccine per bird subcutaneous route in the back of the neck | Laying hens of 12 to 14 weeks and breeders of 14 to 18 weeks of age, previous immunization done with a minimum of two applications of NDV live and IBV |
| 8 | Emulmax AI+ ND | NDV P05 and Avian Influenza subtype H5N2 | SANFER IASA | Inactive vaccine (in oil) | Administer 0.5 ml per bird subcutaneous route in the back of the neck | For broilers, replacement chicks, commercial layers, breeders light and heavy, parents, ducks and turkeys |
| 9 | Emulmax-C AI+ND | NDV P05 and Avian Influenza subtype H5N2 | SANFER IASA | Inactive vaccine (in oil) | Administer 0.2 ml per bird subcutaneous route in the back of the neck | This is a concentrated vaccine for be administer in 1-day-old birds. |
| 10 | Emulmax-C IBH-ND | NDV P05 and Fowl Adenovirus serotype 4 strains SHP-IAP/94, SHP-IAP/95 | SANFER IASA | Inactive vaccine (in oil) | Administer subcutaneous or intramuscular  Broiler: Administer 0.2 ml per bird at 10 to 14 days of age. Breeders: Administer 0.2ml per bird. For bird of 1 kg or more administer 0.4 ml | Two vaccinations before the egg laying period start. |
| 11 | Avinew | NDV VG/GA | Merial  (Now Boehringer Ingelheim) | Live vaccine Freeze-dried | Resuspend in tri-distilled water and administer by spray , drink water or eye drop | For broilers, replacements and breeders |
| 12 | Newcastle B1 | NDV B1 | Merial  (Now Boehringer Ingelheim) | Live vaccine Freeze-dried | Resuspend in tri-distilled water and administer by spray , drinking water or eye drop | For broilers, replacements and breeders |
| 13 | Newcastle LaSota | NDV LaSota | Merial  (Now Boehringer Ingelheim) | Live vaccine Freeze-dried | Resuspend in tri-distilled water and administer by spray, drinking water or eye drop | For broilers, replacements and breeders |
| 14 | Newcastle Bronchitis | NDV LaSota and Infectious bronchitis virus (Massachusetts) | Merial  (Now Boehringer Ingelheim) | Live vaccine Freeze-dried | Re-suspend in 700ml sterile distilled water per vial and administer using spray | For be administering at 1-day-old birds. |
| 15 | AVA-BRON-H N63 | NDV LaSota N63 and Infectious Bronchitis (strain Holland modified from strain Massachusetts) | MSD Animal health | Live vaccine Freeze-dried | Administer by drinking water or eye drop | For vaccination before 16 weeks of age |
| 16 | Combovac 30**®** | NDV Strain B1 (Clone 30) and IBV (Strains Connaught and Connecticut) | MSD Animal health | Live vaccine Freeze-dried | Administer by aspersion with large drop, in the drinking water, or eye drop | Recommended for first vaccination and revaccination in broilers, replacements and breeders after the first week of age |
| **17** | **NOBILIS® Ma5 CLONE 30** | NDV Clone 30, and IBV Serotype Massachusetts Strain Ma5 | MSD Animal health | Live vaccine Freeze-dried | Administer by aspersion with large drop, in the drinking water, or eye drop | Recommended for first vaccination and revaccination in broilers, replacements and breeders after the first week of age |
| **18** | **NOBILIS® ND C2 M** | NDV C2 type B1 and IBV Massachusetts B48 | MSD Animal health | Live vaccine Freeze-dried | Administer by aspersion, with large drop, in the drinking water, or eye drop | For broilers, replacements and breeders at 1-day-old.  In NDV endemic zones revaccinate after 12-days old. |
| 19 | NOBILIS® ND C2 MC | NDV C2 type B1 and IBV Massachusetts B48 and Connecticut | MSD Animal health | Live vaccine Freeze-dried | Administer by aspersion, with large drop in the drinking water, or by eye drop | For broilers, replacements and breeders from day one of age.  In NDV endemic zones revaccinate after 12-days-old. |
| **22** | **NOBILIS® ND CLONE 30** | NDV LaSota Strain Clone 30 | MSD Animal health | Live vaccine Freeze-dried | Administer by aspersion, with large drop in the drinking water, or by eye drop | For 1-day-old birds |
| **23** | **NEWHATCH C2** | NDV type B1 Strain C2 | MSD Animal health | Live vaccine Freeze-dried | Administer by aspersion, ocular or intranasal route. | For 1-day-old birds and older |
| **23** | **NEWHATCH C2-M** | NDV type B1 Strain C2 and IBV Serotype Massachusetts Strain B48 | MSD Animal health | Live vaccine Freeze-dried | Administer by aspersion, ocular or intranasal route. | For 1-day-old birds and older |
| 28 | BREEDERVAC® IV PLUS | IBDV strains standard, variant, Del-A, Del-E and GLS. NDV Clone 30 and IBV M41 (serotype Massachusetts), and Avian Reovirus (Strains 1733 and 2408) | MSD Animal health | Inactive vaccine (in oil) | Administer 0.5 ml subcutaneous. | To be used at 3 to 4 weeks before laying |
| 29 | Newcastle N63 | NDV LaSota N63 | MSD Animal health | Live vaccine Freeze-dried | Administer oral in drinking water, or by eye drop | For broilers at 2-weeks of age or older |
| **30** | **Nolvilis COR4+IB+ND+EDS** | Avibacterium paragallinarum Serotypes A, B and C; IBV (Serotype Massachusetts strain M41); NDV and Egg drop syndrome virus. | MSD Animal health | Inactive vaccine (in oil) | Administer 0.5 ml subcutaneous. | For layers between 14 and 18 weeks of age. For better protection a previous vaccination at 6 to 8 weeks of age is recommended with a 6 weeks interval between vaccinations |
| **34** | **NOBILIS® IB+ND+EDS** | IBV strain M-41 Massachusetts type BI, NDV Clone 30 and BC-14 of Avian Adenovirus | MSD Animal health | Inactive vaccine (in oil) | Inactive vaccine  (in oil) | At 3 to 4 weeks before laying. For Newcastle disease and Bronchitis several previous vaccinations with live virus is recommended. |
|  | **NOBILIS® IB+ND INAC** | IBV strain M-41 Massachusetts type BI, NDV Clone 30 | MSD Animal health | Inactive vaccine (in oil) | Inactive vaccine  (in oil) | For layers at between 10-18-weeks-old |
| **36** | **NOBILIS® NEWCASTRE BROILER INAC** | NDV clone 30 | MSD Animal health | Inactive vaccine (in oil) | Administer 0.25 ml subcutaneous route or intramuscular in the breast | In broilers at 8 to 12 days of age, in commercial layers and breeders, revaccinate between 5 and 18 weeks of age. |
|  | FUSION® ND | Vectored vaccine (Recombinant) HVT expressing F protein of Newcastle disease virus (Strain of NDV are not reported) | MSD Animal health | Vectored Live vaccine Freezer cellular solution | Administer 0.2 ml subcutaneous route on neck | Administer in ovo (18-days-old embryos); at 1-day-old chicks. |
|  | FUSION® ND SB | Vectored vaccine (Recombinant) HVT (Serotype 3) expressing F protein of Newcastle disease virus (Strain of NDV are not reported). Additionally, the formula contains HVT-2 strain SB-1. | MSD Animal health | Vectored Live vaccine Freezer cellular solution | Administer 0.2 ml subcutaneous route on neck | Administer in ovo (18-days-old embryos); at 1-day-old chicks. |
| 37 | CEVAC® BROILER ND K | NDV SZ LaSota | CEVA | Inactive vaccine (in oil) | Administer via subcutaneous route or intramuscular | Administer 0.1 ml subcutaneous in broilers, layers and breeders after day 1 of age |
|  | CEVAC® CORYMUNE 7K | *Avibacterium paragallinarum* (Serotypes A, B y C); *Salmonella enteritidis*; NDV Strain LaSota, IBV strain M-41 and EDSV Strain B8/78 | CEVA | Inactive vaccine (in oil) | Administer via subcutaneous route or intramuscular | Layers after week 14 of age. Birds should be immunized 4 weeks before with individual vaccines |
| 38 | CEVAC® ND IB EDS K | NDV LaSota, and IBV Strain M-41 and Egg drop syndrome virus strain B8/78 | CEVA | Inactive vaccine (in oil) | Administer 0.5 ml subcutaneous or intramuscular | For replacement, breeders and layers previously immunized with live vaccine against IBV and NDV |
| 39 | CEVAC® ND IB K | NDV LaSota and IBV Strain M-41 | CEVA | Inactive vaccine (in oil) | Administer 0.5 ml subcutaneous route at neck | For birds previously immunized with live vaccines. Re-vaccinate at 3 to 4 weeks after first vaccination. Breeders and layers at 16 and 20 weeks of age. |
| 40 | CEVAC® NEW L | NDV LaSota | CEVA | Live vaccine Freeze-dried | Administer by ocular route or drinking water | For birds after of 4-day-old birds and revaccination 3 to 4 weeks later. |
| 41 | CEVAC® VITABRON L | NDV Strain PHY.LMV.42 and IBV Strain H120 (Massachusetts) | CEVA | Live vaccine Freeze-dried | Administer by ocular route or drinking water | For 1-day-old birds or older |
| 42 | CEVAC® VITAPEST L | NDV PHY.LMV.42 | CEVA | Freeze-dried | Administer by the ocular route after day one of age. Drop in the eye: 0.03 ml  Aspersion: from 0.2 a 0.3 ml | At day one or for re-vaccination at 3 to 4 weeks. In replacement birds for the second vaccination at 10 to 12 weeks of age |
| 43 | MAXIMUNE® 8 | IBDV Standard and variant A and E and Avian Reovirus | CEVA | Emulsified inactivated virus | Administer 0.5 ml subcutaneous | For breeders of at least 18 weeks of age. |
|  | CEVAC® VITAPEST | NDV Strain PHY.LMV.42 | CEVA | Freeze-dried | Administer by ocular route or drinking water | For 1-day-old birds or older |
|  | MAXIMUNE® 8 | Infectious bursal disease virus (IBDV) Standard and variant strains (Delaware A, Delaware E) NDV Strain LaSota, IBV Strains H52, Mass 41; and Avian Reovirus Strains sil 33, 2408 and 55412. | CEVA | Emulsified | Administer 0.5 ml. subcutaneous route | For bird after 18-weeks-old |
| 44 | VECTORMUNE ® HVT NDV | Recombinant HVT serotype 3 (vector) expressing F protein of NDV | CEVA | Vectored live vaccine Freezer cellular solution | in-ovo or 1-day old birds | For 1-day-old birds of in ovo at 18 days post incubation |
| **45** | **Volvac ND LaSota MLV** | Modified NDV LaSota | Boehringer Ingelheim | Live vaccine Freeze-dried | Administer by spray, oral in drinking water or eye drop | Birds as first vaccine. |
| **46** | **Volvac ND+ IB MLV** | Modified NDV LaSota and IBV serotype Massachusetts | Boehringer Ingelheim | Live vaccine Freeze-dried | Administer by ocular route, drinking water and spray | For broilers, replacement and layers before 21-days-old |
| **47** | **Volvac ND+FC KV** | Modified NDV LaSota and three *Pasteurella multocida* (FC) serotypes | Boehringer Ingelheim | Inactive vaccine (in oil) | Administer 0.5 ml subcutaneous route | For broilers, layer replacement, layers and breeders between 4 and 18 weeks of age. Two applications separated by 8 to 10 weeks. |
| **48** | **Volvac AI+ND KV** | AIV subtype H5N2 and NDV LaSota | Boehringer Ingelheim | Inactive vaccine (in oil) | Administer 0.5 ml subcutaneous route | To be used in layers before 14 to 16 weeks of age. |
| **49** | **Volvac AC Plus+ ND+ IB+ EDS KV** | NDV LaSota; Infectious coryza (serotype A, B and C), IBV Serotype Massachusetts strain M-41; and Egg drop syndrome virus strain 127 | Boehringer Ingelheim | Inactive vaccine (in oil) | Administer 0.5 ml. subcutaneous route | To be used in layers before 14 to 16 weeks of age. |
| **50** | **Volvac ND KV** | NDV LaSota | Boehringer Ingelheim | Inactive vaccine (in oil) | Administer 0.5 ml. subcutaneous route | To be used in healthy birds for prevention of Newcastle disease |
| **51** | **Volvac ND+ IB+EDS KV** | NDV LaSota, IBV serotype Massachusetts and Egg drop syndrome virus strain 127 | Boehringer Ingelheim | Inactive vaccine (in oil) | Administer 0.5 ml. subcutaneous route | To be used in healthy birds for prevention of Newcastle disease, infectious bronchitis and egg drop syndrome |
| **52** | **Volvac ND Conc. KV** | NDV LaSota | Boehringer Ingelheim | Inactive vaccine (in oil) | Administer 0.2 ml. subcutaneous route | To be used in healthy birds for prevention of Newcastle disease |
| **53** | **Volvac ND+IB KV** | NDV LaSota and IBV Serotype Massachusetts. | Boehringer Ingelheim | Inactive vaccine (in oil) | Administer 0.5 ml. subcutaneous route | To be used in healthy birds for prevention of Newcastle disease and infectious bronchitis |
| **54** | **Volvac AC Plus+ND+IB KV** | Avibacterium paragallinarum (AC) serotype A, B and C NDV LaSota, IBV serotype Massachusetts | Boehringer Ingelheim | Inactive vaccine (in oil) | Administer 0.5 ml. subcutaneous route | To be used in healthy birds for prevention of infectious coryza, Newcastle disease and infectious bronchitis |
| **55** | **Volvac Gallibacterium+ND+FC KV** | NDV LaSota, Gallibacterium anatis (Ga); Pasteurella multocida( FC). | Boehringer Ingelheim | Inactive vaccine (in oil) | Administer 0.5 ml. subcutaneous route | For replacement, layers and breeders between 4 and 18 weeks of age |
| **56** | **Volvac AI+ND CONC KV** | Avian Influenza subtype H5N2 and NDV LaSota. | Boehringer Ingelheim | Inactive vaccine (in oil) | Administer 0.5 ml subcutaneous route | To be used at 8 to 10 days of age and at 3 to 4 weeks using live NDV. |
| **58** | **Newcastle K** | NDV Kimber | ZOETIS | Inactive vaccine (in oil) | Administer 0.5 ml. Intramuscular or subcutaneous route using aseptic measures | For replacement, layers and breeders after initial vaccination |
| 59 | PRO VAC 4 ACL | IBDV Lukert, Viral arthritis strain1133 and 2408, NDV Kimber and IBV strain Mass 41 | ZOETIS | Inactive vaccine (in oil) | Warm up at room temperature, shake for resuspend, inoculate Administer 0.5 ml. subcutaneous route in dorsal median neck region or intramuscular | Require previous immunization against IBDV and Reovirus for weeks previous to this vaccine. |
| 61 | NEWCASTLE B1 + BRON CONN MASS | NDV strain B1, IBV strains Connecticut and Massachusetts | ZOETIS | Live vaccine Freeze-dried | Administer oral, intranasal o intraocular. | One drop per bird at day 1 of age and henceforth. |
| 62 | NEWCASTLE LASOTA | NDV LaSota strain B1 | ZOETIS | Live vaccine Freeze-dried | Administer intranasal, by eye drop or in drinking water. | Administer in 1-day-old birds. For aspersion use only starting from 4 weeks birds of age. |
| 63 | NEWCASTLE LASOTA + BRON MASS | NDV strain LaSota B1; and IBV strain Massachusetts | ZOETIS | Live vaccine Freeze-dried | Administer intranasal, by eye drop or in drinking water | Administer in 1-day-old birds. For aspersion use only starting from 4 weeks birds of age. |
| **64** | **Newcastle B1** | NDV Strain B1 | ZOETIS | Live vaccine Freeze-dried | For ocular or intranasal route by spray or use in drinking water. | For first dose or for revaccination. |
| **65** | **TRIPLE AVIAR** | NDV LaSota Pasteurella multocida A and Pasteurella multocida X 73 | Biozoo | Inactive vaccine (in oil) | Administer intramuscular, 0.5ml on 10 to 30 days of age birds and 1ml on 30 days or older. | For boost vaccination at day 21. |
| **66** | **Triple Aviar Curativa** | NDV LaSota Pasteurella multocida A and Pasteurella multocida X 73 | Biozoo | Inactive vaccine (in oil) | Administer intramuscular, 0.5ml on 10 to 30 days of age birds and 1ml on 30 days or older. | Administer first dose at day 10 of age, Boost at day 21 and third application between 16 and 18 weeks of age. |
| **67** | **Newcastle** | NDV LaSota | Biozoo | Live vaccine Freeze-dried | For oral or nasal use resuspend with included diluent | Three applications recommended. Oral vaccination at day 10 of age, first boost at days 18 to 20, second boost between 56 and 63 days of age. For ocular and nasal, first vaccination at day 10 days of age, 2º between18 and 20 weeks and between 3 and 4 months of age |
| **69** | **Newca-mex** | NDV LaSota | AVIMEX | Inactive vaccine (in oil) | Day one use 0.5 ml/bird by subcutaneous route at the neck or intramuscular (breeders and layers). | To vaccinate and revaccinate at day 8 or older. For Broiler use between day 8 and 12. To revaccinate use during 3 to 4 weeks old birds. For layers use two to 3 times during development 6 to 7 weeks and 6 weeks before laying start. |
| **70** | **Newca-mex concentrada** | NDV LaSota | AVIMEX | Inactive vaccine (in oil) | Day one use 0.2ml/bird by subcutaneous route at the neck or intramuscular (breeders and layers). | On birds 8 day or older administer between 0.3 and 0.5ml. |
| **71** | **Newcastle Bronchitis** | NDV LaSota. IBV  BI, Massachusetts and Connecticut | AVIMEX | Inactive vaccine (in oil) | Administer 0.5 ml/bird via subcutaneous route in neck or intramuscular in breast (for breeders and layers). | To vaccinate healthy birds at day 8 or older. To revaccinate 2 to 3 times during development and 6 weeks before layer production start. |
| **72** | **Newcastle Bronchitis-EDS** | NDV LaSota. IBV  serotypes Massachusetts and Connecticut  and Egg drop syndrome virus strain JPA | AVIMEX | Inactive vaccine (in oil) | Administer 0.5 ml/bird via subcutaneous route in neck or intramuscular in breast (for breeders and layers). | To vaccinate twice. Use with live NDV and IBV vaccines |
| **73** | **Newcastle bronchitis Hepatitis** | NDV strain LaSota; IBV strains  Massachusetts and Connecticut and Fowl Adenovirus group 1 serotype 4 | AVIMEX | Inactive vaccine (in oil) | Administer 0.5 ml/bird via subcutaneous route in neck or intramuscular in breast (for breeders and layers). | To vaccinate healthy birds at day 8 or older. To revaccinate 2 to 3 times during development and 6 weeks before layer production start. |
| **74** | **Newcastle-*E.coli*** | NDV LaSota and  E. coli serotype O1, O2 and O78 | AVIMEX | Inactive vaccine (in oil) | Administer 0.5 ml/bird via subcutaneous route in neck or intramuscular in breast (for breeders and layers). | To vaccinate healthy birds at day 8 or older. In Broilers **a single application at day 8 to 12 of age. Always use this vaccine together with live vaccines against NDV.** |
| **75** | **Newcastle-Coriza** | NDV LaSota and pure cultures of Avibacterium (Haemophilus paragallinarum) serotypes A, B and C | AVIMEX | Inactive vaccine (in oil) | Administer 0.5 ml/bird via subcutaneous route in neck or intramuscular in breast (for breeders and layers). | To vaccinate healthy birds at day 8 or older. In Broilers **a single application at day 8 to 12 of age. For layers** revaccinate 2 to 3 times during development and 6 weeks before layer production start. |
| **76** | **Newcastle EDS** | NDV LaSota  and egg drop syndrome virus strain JPA | AVIMEX | Inactive vaccine (in oil) | Administer 0.5 ml/bird via subcutaneous route in neck or intramuscular in breast (for breeders and layers). | For layers or breeders healthy during development. **For layers** revaccinate 2 to 3 times during development and 6 weeks before layer production start.  To be used always with live vaccines |
| **77** | **Newcastle-Hepatitis** | NDV LaSota  and Fowl Adenovirus group 1 serotype 4 | AVIMEX | Inactive vaccine (in oil) | Administer 0.5 ml/bird via subcutaneous route in neck or intramuscular in breast (for breeders and layers). | To vaccinate healthy birds at day 8 or older. In Broilers **a single application at day 8 to 12 of age. For layers** revaccinate 2 to 3 times during development every 6 weeks and 6 weeks before layer production start. |
| **78** | **Newcastle influenza** | NDV LaSota and  AIV subtype H5N2 | AVIMEX | Inactive vaccine (in oil) | Administer 0.5 ml/bird via subcutaneous route in neck or intramuscular in breast (for breeders and layers). | To vaccinate healthy birds at day 8 or older. In Broilers **a single application at day 8 to 12 of age. For layers** revaccinate 2 to 3 times during development every 6 weeks and 6 weeks before layer production start. |
| **79** | **Newcastle influenza Hepatitis** | NDV strain LaSota; avian influenza virus subtype H5N2 and Fowl Adenovirus serotype 4 | AVIMEX | Inactive vaccine (in oil) | Administer 0.5 ml/bird via subcutaneous route in neck or intramuscular in breast (for breeders and layers). | To vaccinate healthy birds at day 8 or older. In Broilers **a single application at day 8 to 12 of age. For layers** revaccinate 2 to 3 times during development every 6 weeks and 6 weeks before layer production start. |
| **80** | **LaSota** | NDV LaSota | AVIMEX | Live vaccine  Freeze-dried | Administer by ocular, spray or in drinking water. | To vaccinate and revaccinate healthy birds at day one and henceforth. For broilers it is recommended to vaccine in incubators at day one with two revaccinations at days 8-12 and 21-28 days of age. For replacements 3 to 4 applications **before laying start every 3 to 5 weeks.** Can be used simultaneously with emulsified vaccines. During egg production administer every 2 to 3 months if necessary. |
| 81 | Innovac rND-H5 | Vectored vaccine based in NDV strain B1 that express HA from influenza virus subtype H5 | AVIMEX | Vectored Live vaccine  Freeze-dried | Administer ocular, aspersion or in drinking water. | To vaccinate and revaccinate healthy birds at day one and henceforth. For broilers it is recommended to vaccine in incubators at day one with two revaccinations at days 8-12 and 21-28 days of age. For replacements 3 to 4 applications **before laying start every 3 to 5 weeks.** Can be used simultaneously with emulsified vaccines. During egg production administer every 2 to 3 months if necessary. |
| **82** | **New H5** | Vectored vaccine based in NDV strain LaSota expressing gene HA of avian influenza virus subtype H5 | AVIMEX | Vectored Live vaccine  Freeze-dried | Administer ocular, aspersion or in drinking water. | For healthy broilers of 12 days of age and henceforth with revaccination at **21 to 28 days of age.** For replacements is recommended 2 to 3 applications **before laying start every 3 to 5 weeks.** Can be used simultaneously with emulsified vaccines. During egg production administer every 2 to 3 months if necessary. |
| **83** | **Hepatitis- Newcastle concentrada** | NDV LaSota and  Fowl Adenovirus group 1 serotype 4 | AVIMEX | Inactive vaccine (in oil) | Administer between 0.2 and 0.5 ml/bird | To vaccinate and revaccinate healthy birds at day one and henceforth. **In broilers,** the vaccination program can be modified according to the age of the birds at marketing. When birds will be used before day 35 an application at day of age is acceptable. When birds are used after day 35, an application at 8 days of age is recommended. For layers or **breeders**, vaccinated 2 to 3 during development separated every 6 to 7 and up to 6 months before egg production start. This must be accompanied with live NDV vaccines. |
| **84** | **NEWXXITEK** | Vectored vaccine (Recombinant) HVT (Serotype 3) expressing gene F of genotyp4 IV Velogenic Texas virus | Merial (now Boehringer Ingelheim Animal Health) | Vectored live vaccine Freezer cellular solution |  | \| Administer in ovo  (18-days-old embryos);  at 1-day-old chicks. \| \| --- \| |

Table 2. Oubreaks in Latin America
